# Predicting food crises using news streams

**DOI:** 10.1126/sciadv.abm3449

**Published:** 2023-03-03

**Authors:** Ananth Balashankar, Lakshminarayanan Subramanian, Samuel P. Fraiberger

**Affiliations:** ^1^ Department of Computer Science, New York University, New York, NY, USA.; ^2^ Department of Population Health, NYU Grossman School of Medicine, New York, NY, USA.; ^3^ Development Data Group, World Bank, Washington, DC, USA.; ^4^ Connection Science, Massachusetts Institute of Technology, Cambridge, MA, USA.

## Abstract

Anticipating food crisis outbreaks is crucial to efficiently allocate emergency relief and reduce human suffering. However, existing predictive models rely on risk measures that are often delayed, outdated, or incomplete. Using the text of 11.2 million news articles focused on food-insecure countries and published between 1980 and 2020, we leverage recent advances in deep learning to extract high-frequency precursors to food crises that are both interpretable and validated by traditional risk indicators. We demonstrate that over the period from July 2009 to July 2020 and across 21 food-insecure countries, news indicators substantially improve the district-level predictions of food insecurity up to 12 months ahead relative to baseline models that do not include text information. These results could have profound implications on how humanitarian aid gets allocated and open previously unexplored avenues for machine learning to improve decision-making in data-scarce environments.

## Teaser

Text indicators extracted from news articles using machine learning help predict food crises in data-scarce environments.

## INTRODUCTION

Food insecurity continues to threaten the lives of hundreds of millions of people around the world today. According to the Food and Agriculture Organization of the United Nations, the number of undernourished increased from 624 million people in 2014 to 688 million in 2019 (*1*). There is considerable evidence that quickly responding to emerging risks of food insecurity saves lives and lowers humanitarian costs (*2*), which leads aid agencies to resort to early warning systems to decide when and where to deploy emergency relief (*3*). While risk factors are well-established (*4*–*7*), ranging from conflicts to pests, weather shocks, and migration, delayed or infrequent measurements of these factors typically impede early warning systems’ ability to promptly anticipate food crises (*8*). Furthermore, food-insecure countries often lack the capacity to systematically measure risk factors, generating data gaps (*9*).

Against this backdrop, the past decade has seen an explosion in the availability of vast repositories of digital data, from satellite imagery to call detailed records, which are increasingly being analyzed to address socioeconomic challenges (*10*, *11*). Encouraged by these approaches, we take advantage of recent advances in deep learning and natural language processing to extract anticipatory signals of food insecurity episodes from the text of a large corpus of news articles. We postulate that the forces triggering a food crisis are mentioned in the news before being observable with traditional risk measures. Unlike existing food insecurity early warning systems, the articles we collect are published on a daily basis, allowing us to generate high-frequency forecasts (*12*). News aggregators provide access to media articles curated in a transparent manner going back several decades, enabling the analysis of long-time series of news streams (*13*). Lastly, authoritative media sources such as *BBC* or *Reuters* have a long-standing reputation for providing trustworthy information on local contexts, suited to produce disaggregated forecasts (*14*).

## RESULTS

### Context of the study

Our study focuses on predicting the Integrated Phase Classification (IPC) of food insecurity published by the Famine Early Warning Systems Network (FEWS NET) (https://fews.net). This classification is available at the district level across 37 food-insecure countries in Africa, Asia, and Latin America and was reported four times per year between 2009 and 2015 and three times per year thereafter. Food insecurity is classified according to an ordinal scale composed of five phases: minimal, stressed, crisis, emergency, and famine (see Materials and Methods). To demonstrate the predictive value of text information contained in news articles, we collect a dataset from the news aggregator Factiva containing the 11.2 million articles focusing on the countries covered by the IPC dataset and published between 1980 and 2020 (see Materials and Methods).

### Text features selection

We then develop a methodology based on semantic role labeling to uncover text features related to food insecurity. We start from 13 seed key phrases semantically close to food insecurity (fig. S3A), and we use a frame-semantic parser to extract semantic causes of food insecurity appearing in the same frame as one of the seeds (see Materials and Methods). For example, when the parser examines the sentence “Famine may return to some parts of the country, with the eastern Pibor county, where floods and pests have ravaged crops, at particular risk,” it detects that “floods” and “pests,” both being established causes of food insecurity, are mentioned in the same semantic frame as the seed “famine” (*15*, *16*). We apply this method to our corpus of news, and we uncover 1062 text features consisting of unigrams, bigrams, and trigrams occurring in the same semantic frame as a seed key phrase. To ensure that this approach captures a wide range of semantic causes of food insecurity recognized both by journalists and by experts, we repeat the same procedure on 93 manually selected peer-reviewed studies and books on food insecurity, which reveals 149 additional text features (see Materials and Methods).

We then expand this initial list of 1211 features by considering candidate features mentioned in the news and semantically close to an initial feature (*17*, *18*), obtaining 738 features (see Materials and Methods). Lastly, to drop any irrelevant feature that might have accidentally been picked up, we first convert each of the 1949 text features into a district-level time series of the proportion of monthly news articles mentioning both the feature and the district (“news factor”). We then run Granger causality tests using a temporal train-validation split over the period from July 2009 to July 2010 and discard news factors that fail to Granger-cause the IPC phase, which leads to a final set of 167 text features (see Materials and Methods).

**Fig. 1.**
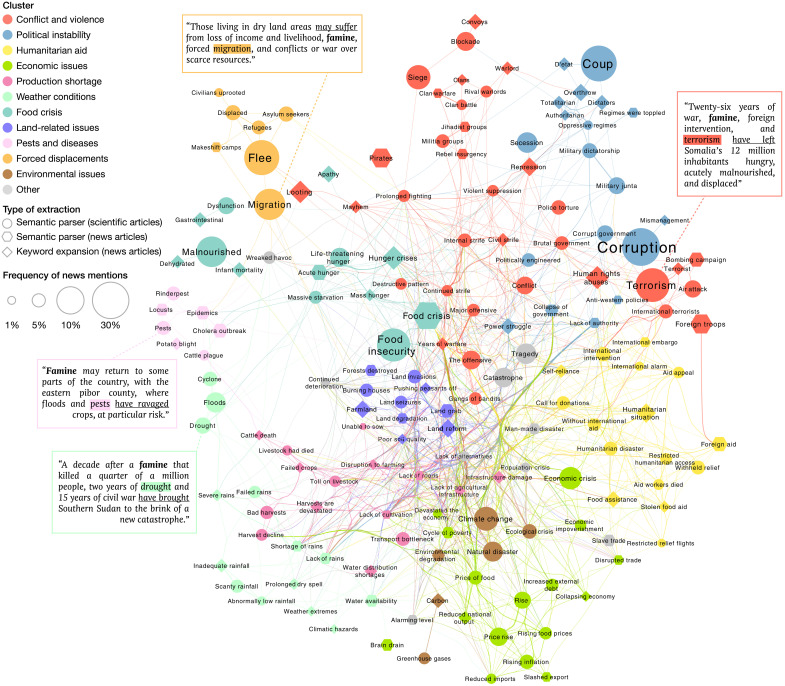
Uncovering text features related to food insecurity. Starting from a handcrafted list of 13 seed key phrases related to food insecurity, we use a frame-semantic parser to extract from scientific (circles) and news (hexagons) articles unigrams, bigrams, and trigrams (“text features”) associated with one of the seeds (*15*, *16*). Each box contains an example of a sentence in which the parser detects a text feature (highlighted in color) occurring in the same semantic frame as the seed “famine” (in bold) and a causal link (underlined). We expand this original list with text features from news articles (diamond) that are semantically close to an original text feature according to their word mover’s distance (*17*, *18*). Text features for which the proportion of monthly local news mentions fails to predict the IPC classification of food insecurity are discarded, leading to a final set of 167 features grouped into 12 clusters based on their semantic similarity and mapped onto a network. A node’s size is proportional to its text feature’s frequency in news articles mentioning seeds, and an edge’s width encodes the semantic proximity between its end nodes’ text features. A force-directed algorithm determines each node’s position, leading nodes representing semantically similar text features to appear close to one another.

To assess the content validity of these 167 features, we partition them into 12 semantically distinct clusters. Text features belonging to the same clusters co-occur in the news about twice as frequently as those in different clusters—the average pairwise correlation between news factors being 69.9 and 34.9%, respectively—which provides support to our partitioning (Fig. 1 and fig. S5). Furthermore, 9 of 12 clusters are related to known causes of food insecurity—“conflict and violence,” “political instability,” “economic issues,” “production shortage,” “weather conditions,” “land-related issues,” “pests and diseases,” “forced displacements,” and “environmental issues”—accounting for 92% of the articles in which text features are mentioned. The remaining three clusters include terms related to “food crisis,” “humanitarian aid,” and “other” negative terms unspecific to food insecurity. These results establish the content validity of our text features by demonstrating that they encompass a wide range of causes of food insecurity (see Materials and Methods).

**Fig. 2.**
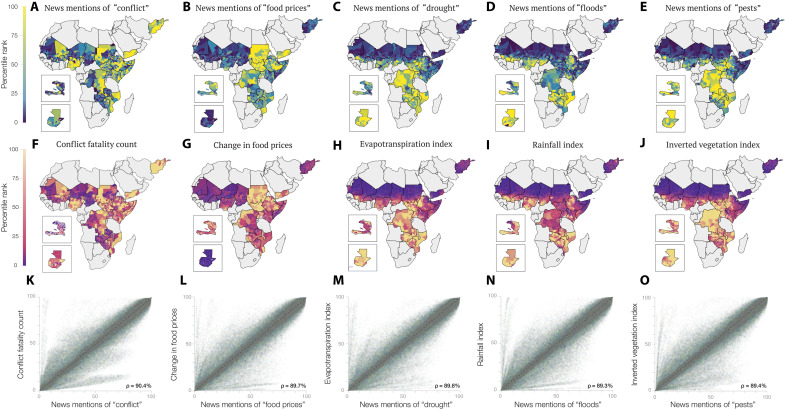
Validating news indicators of food insecurity. Five traditional food insecurity risk factors—a conflict fatality count, the change in food prices, an evapotranspiration index, a rainfall index, and an inverted vegetation index—are available for 21 food-insecure countries during the period from July 2009 to July 2020. We associate with each traditional risk factor the news factor with which it has the highest Spearman correlation across districts and months. (**A** to **J**) The spatial distribution of each traditional risk factor and its associated news factor in October 2017 and (**K** to **O**) a scatterplot of a risk factor (*y* axis) and its associated news factor (*x* axis), which reveals a high Spearman correlation and provides validity to our news constructs. All the values are reported in percentile rank.

We then explore the relationship between news factors and traditional food insecurity risk factors (Fig. 2). We use a comprehensive dataset of traditional risk factors from the literature—a conflict fatality count, the change in food prices, an evapotranspiration index, a rainfall index, and an inverted vegetation index—covering 21 food-insecure countries over the period from July 2009 to July 2020 (fig. S6). We then associate with each traditional factor the news factor with which it has the highest Spearman correlation across districts and months. We find that the conflict fatality count, change in food prices, evapotranspiration index, rainfall index, and inverted vegetation index are most strongly correlated to news mentions of “conflict,” “food prices,” “drought,” “floods,” and “pests,” respectively (ρ > 0.89), thereby providing additional validity to our news constructs. Together, these results indicate that our procedure allows us to uncover text features that are interpretable and validated by traditional risk indicators.

### Predicting food insecurity

**Fig. 3.**
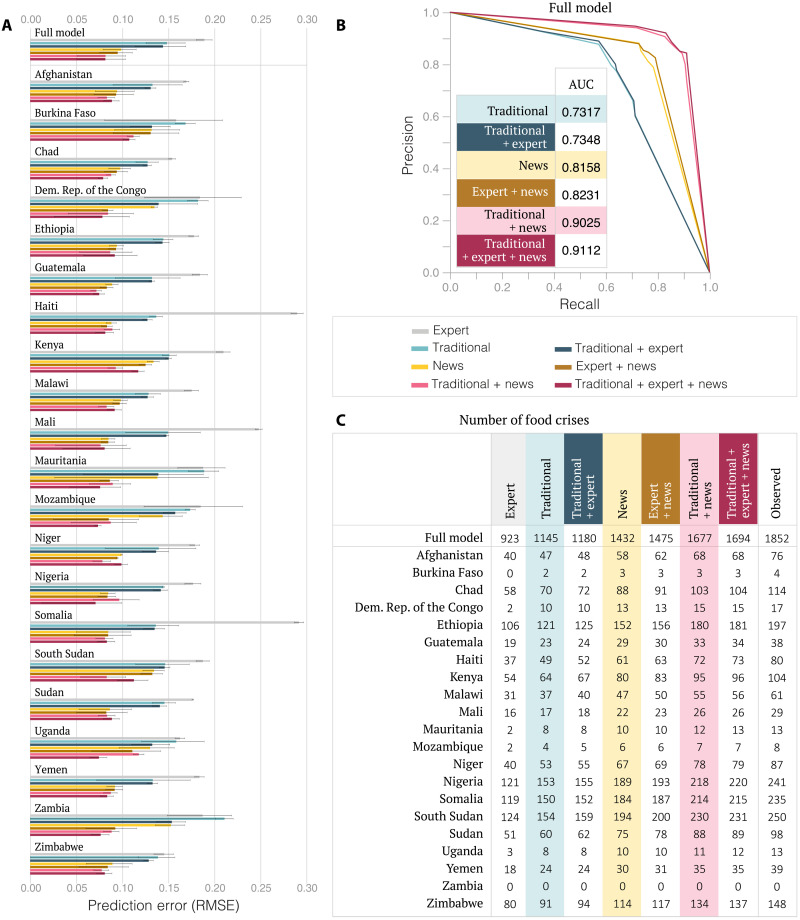
Predicting food insecurity. (**A**) District-level predictions of the IPC classification of food insecurity 3 months ahead obtained from expert forecasts and from six random forest models estimated using observations from 21 countries over the period from July 2009 to July 2020. We show the cross-validation RMSE and its 95% confidence interval on the entire test set (full model) and for each country separately. While the traditional model outperforms the expert forecasts by 22%, the news and traditional + news models further reduce the forecast error of the traditional model by 34 and 45%, respectively. (**B**) Classification of food crisis outbreaks, which corresponds to the dates when the IPC phase raises to a value of 3 or more for at least two consecutive periods. We convert the estimated random forest regressions into binary classifiers of outbreaks using a classification threshold. By varying the threshold, we construct a model’s Pareto front (full line) and we report the area under the curve (AUC). Consistent with the results from the regression models, we find that the AUC of the news model is larger than that of the traditional model, that the traditional + news model leads to better classification performances than each model individually, and that including expert forecasts has almost no impact on any of the models. (**C**) Number of outbreaks observed in the test set (white) and predicted by each model for an 80% precision, showing that the news and traditional + news models lead to an increase in recall of 25 and 46%, respectively, relative to the traditional model. With a precision and a recall equal to 0.71 and 0.51, respectively, the expert forecasts are outperformed by all the predictive models.

Next, we demonstrate that news factors help improve existing predictions of food insecurity. Together with their latest measure of the IPC phase, FEWS NET publishes expert forecasts of next period’s values, which are closely scrutinized by policy-makers. As a first baseline, we therefore estimate the performance of experts at predicting the IPC phase next period. Our second baseline is a random forest regression model of the IPC phase 3 months ahead using the traditional factors described in Fig. 2 along with time-invariant risk factors (*19*): the population count, district size, terrain ruggedness, and share of agricultural land use (“traditional model”) (see Materials and Methods). Observations in the traditional model are measured at the district-quarter level across 21 countries, and the model is estimated using temporal cross-validation over the period from July 2009 to July 2020 (fig. S7). In line with previous research (*8*), we find that the traditional model substantially outperforms the expert forecasts, with out-of-sample root mean square errors (RMSEs) equal to 0.1486 and 0.1892 (Fig. 3A and fig. S8).

We then compare the predictive performance of the traditional model to that of the same random forest in which we substitute traditional factors with news factors (“news model”), which leads to a drop in RMSE to 0.0989 (Fig. 3A and fig. S8). While traditional risk factors are costly and time-consuming to collect, these results indicate that news factors could not only serve as a cost-effective substitute but also lead to better performances. Furthermore, when we combine both traditional and news factors into the same random forest (“traditional + news model”), the RMSE is further reduced to 0.0819, proving that news indicators of food insecurity also serve as a complement to traditional risk indicators (Fig. 3A and fig. S8). Additional robustness checks demonstrate that all the steps of our feature selection approach to find relevant key phrases are necessary to obtain these large reductions in RMSE (fig. S10). Incorporating news factors even improves the predictions of the traditional model up to 12 months into the future (fig. S8). By contrast, including expert forecasts has no significant incidence on the RMSE of either the traditional model (Diebold-Mariano test, *P* = 0.1104), the news model (Diebold-Mariano test, *P* = 0.1311), or the traditional + news model (Diebold-Mariano test, *P* = 0.1528) (Fig. 3A and fig. S8).

While the traditional model considerably surpasses the expert forecasts, the latter can serve as a reasonable baseline for the 16 countries covered by FEWS NET in which traditional risk indicators are not available from previous research (fig. S8). We find that the news model also outperforms the expert forecasts in these 16 countries and that incorporating expert forecasts into the news model has no significant impact on its RMSE (Diebold-Mariano test, *P* = 0.1211).

Together, these results demonstrate that news factors consistently improve existing predictions of food security at the district level. However, we observe that news-based forecasts are substantially more accurate in regions with higher news coverage (Fig. 3A and fig. S11), suggesting that these forecasts could be affected by publishers’ decisions to preferentially report on specific regions or events or even by authoritarian censorship of the press. To shed light on how changes in news coverage could affect forecast performances, we simulate the shutdown of the largest news sources in our dataset and its impact on the news-based forecasts (fig. S10). We find that removing all news articles published by “All Africa Global Media,” the largest news source representing 18.4% of all articles, from the traditional + news model only increases its RMSE by 16.5%, from 0.0819 to 0.0953. When removing all articles published by the 10 largest news sources that represent 38.4% of all articles, the RMSE deteriorates by 39%, from 0.0819 to 0.1137. Even under this extreme scenario, incorporating news factors into the traditional model still leads to a large improvement in its RMSE, indicating that the model retains most of its predictive power from the numerous smaller news sources.

### Predicting food crisis outbreaks

To put these results into perspective, we quantify the role played by news factors in specifically improving the prediction of food crisis outbreaks. We define a food crisis outbreak as the date when the IPC phase raises to a value of 3 or more for at least two consecutive periods, an event of utmost importance to disaster relief organizations deciding when and where to allocate emergency food assistance (*8*). We convert each of the previously estimated models into a binary classifier of crisis outbreaks and compare their classification performances (see Materials and Methods).

Consistent with the results from the regression models, we find that the area under the precision-recall curve (AUC) of the news model is larger than that of the traditional model, that the traditional + news model leads to better classification performances than each model individually, and that including expert forecasts has almost no impact on any of the models (Fig. 3B). While the AUC offers a threshold-agnostic measure of performance, it also has downsides: It aggregates over regions of the precision-recall curve in which decision-makers would rarely operate, e.g., at a very low precision or a very low recall, and it gives equal weights to false positives and false negatives. We remain agnostic about how to optimally weight type I and type II errors; however, we recognize that failing to predict a food crisis could lead to much more marked consequences than falsely predicting one. It is therefore more relevant to fix a realistic precision level and compare the number of food crisis outbreaks predicted by each model with the actual number of outbreaks observed across test periods.

Fixing precision at 80%, we find that the news factors lead to large gains in recall: the traditional model, news model, and traditional + news model respectively predict 1145, 1432, and 1677 of the 1852 food crisis outbreaks observed across test periods (Fig. 3C). News factors are equally valuable in anticipating the most severe outbreaks: The traditional + news model predicts 43 of the 48 outbreaks during which the IPC phase escalates to a level 4 or 5, while the traditional and the news models respectively only predict 26 and 33 of these outbreaks. Our definition of an outbreak ensures that we can issue warnings before a prolonged crisis phase; however, incorporating news factors into the traditional model improves its performance independently of the number of periods characterizing an outbreak (fig. S10D). In addition, for a wide range of realistic precision values from 60 to 90%, incorporating news factors into the traditional model produces large gains in recall between 30 and 78% (fig. S10D). Together, these results demonstrate that our news indicators accurately anticipate variations in district-level IPC phases and could help dispatch emergency relief up to 12 months ahead of a food crisis.

### Case studies

While machine learning is often criticized for its lack of transparency (*20*), our model’s predictions can easily be interpreted. Focusing on Somalia, South Sudan, and Ethiopia, three of the countries that experienced the highest level of food insecurity in recent decades, we zoom in on specific crisis episodes to elicit which news factors help predict the deterioration of the situation.

**Fig. 4.**
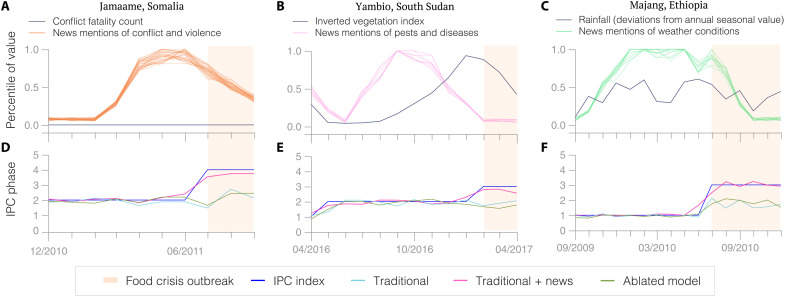
Food crises episodes. (**A** to **C**) We zoom in on three crisis episodes during which news mentions of key phrases related to “conflict and violence” (red), “pests and diseases” (pink), and “weather conditions” (green) would have helped anticipate a food crisis outbreak (mauve shaded area). For each episode, we report each text feature’s proportion of monthly local news mentions and the associated traditional risk factor (black). All the values are reported in percentiles. (**D** to **F**) We also report the time series of the IPC phase (blue) and its predicted value using the traditional (turquoise), ablated (khaki), and traditional + news (red) model. While traditional risk factors fail to provide a warning signal in a timely fashion, news factors peak before each crisis outbreak, leading the traditional + news model to accurately predict the outbreak, whereas the traditional and ablated models fail to predict it.

The first episode that we analyze happened in 2011 in Somalia, where the combination of a drought, rising food prices, forced displacement, and a sustained conflict led to the worst famine of the 21st century (*21*). In particular, the district of Jamaame evolved from an IPC phase 2 during the first half of 2011 to a phase 4 by July 2011, following intensifying violence in the southern part of the country. While the proportion of news articles mentioning both Jamaame and terms included in the “conflict and violence” cluster started raising 5 months before the change in the IPC phase, the conflict fatality count did not record any death in the district until the summer of 2012, highlighting that news factors capture relevant dimensions of civil insecurity which are missing from traditional conflict indicators (Fig. 4, A and D).

The second episode that we focus on occurred in 2016 when a fall armyworm spread across 20 countries in Africa, decimating large quantities of crops (*22*). The worm was first reported in early 2016, and by September, the proportion of news mentioning both the Yambio county in South Sudan and text features included in the “pests and diseases” cluster had peaked, 4 months before the inverted vegetation index peaking, and 5 months ahead of the IPC phase raising from 2 to 3 (Fig. 4, B and E). Although pest infestations are indirectly measured through vegetation indices, their damage on crops are typically only reflected in vegetation greenness once the food security of neighboring populations has begun to deteriorate, strengthening the importance of measuring anticipatory signals from the news.

Last, the last episode of our study took place in 2009 in Ethiopia when it experienced one of its driest years of the past 50 years, wreaking havoc on food production (*23*). Seasonality-adjusted levels of precipitation in the Majang district were 2.3 SDs below their historical average in September 2009 before reverting to their mean at the beginning of 2010. While the prolonged effect of this extreme drought was not well captured by the precipitation index, the proportion of news mentioning both Majang and terms contained in the “weather conditions” cluster started increasing in late 2009 and remained close to its peak until July 2010 when the IPC phase increased from 1 to 3, suggesting that news indices are also better suited to anticipate a drought-related food crisis (Fig. 4, C and F).

To quantify the role played by news factors in driving our predictions during these episodes, we reestimate the traditional + news model after having removed the cluster of news factors containing terms related to “conflict and violence,” “pests and diseases,” and “weather conditions” (“ablated models”). For all three episodes, we find that the traditional + news model can accurately anticipate the change in the IPC phase, whereas both the ablated model and the traditional model fail to predict it (Fig. 4, D to F). In other words, risk factors leading to a food crisis are better anticipated by news indicators than by traditional ones, which can be incomplete, delayed, or outdated, and our model enables us to explicitly interpret predictions of food crisis outbreaks by linking them to variations in news mentions of the underlying causes of an upcoming outbreak.

## DISCUSSION

Although the drivers of food insecurity are well known, early warning systems relying on high-frequency measurements of these factors are still lacking. The data-driven approach described in this paper could drastically improve the prediction of food crisis outbreaks up to 12 months ahead of time using real-time news streams and a predictive model that is simple to interpret and explain to policy-makers. Development practitioners working for humanitarian organizations such as the World Food Program could use the predictions of our model to help prioritize the allocation of emergency food assistance across vulnerable regions in a principled way, allowing for a more effective preparedness and a reduction in human suffering when a crisis hits. Early warnings cannot address all of the sources of delay in emergency responses; however, it can mitigate it by increasing the cost of inaction for governments and the international community (*24*). While our study only focuses on news articles in English, future work incorporating local languages into our framework could potentially improve the predictive performance of our model even further. Beyond the context of food insecurity, our approach for selecting news indicators that are predictive, valid, and interpretable could be extended to other domains, from disease surveillance to the impact of climate change, where big data and machine learning are being used to predict policy outcomes in data-scarce environments (*13*, *25*, *26*).

## MATERIALS AND METHODS

### Food insecurity dataset

Our dataset on food insecurity comes from the FEWS NET. Food insecurity is classified into five phases following the IPC framework: (i) minimal, (ii) stressed, (iii) crisis, (iv) emergency, and (v) famine. The IPC classification is generated from a range of indicators such as the quantity and quality of food consumption, food acquisition strategies, distribution of body mass indices, excess mortality due to food consumption deficit, presence of humanitarian assistance, etc. Experts from various fields assess empirical evidence on these outcomes and convert them into an IPC phase using an analytical framework defined by the IPC guidelines (*27*). This enables the comparison of food insecurity levels across time and geographies in a standardized way. The FEWS NET classification of food insecurity is available at the district level for 37 countries (fig. S1). It was established four times per year from 2009 to 2015—in January, April, July, and October—and three times per year thereafter—in February, June, and October.

### News articles dataset

Our dataset of news articles comes from Factiva, a digital archive of global news content. Factiva aggregates more than 33,000 news resources from 200 countries in 28 languages. Each news article is tagged with geographic region codes to ascertain its relevance to a specific country. We collect the text of the 11.2 million articles in English obtained from 5421 news sources and tagged with at least 1 of the 37 countries covered by FEWS NET (fig. S2). Twelve percent of articles focus on non-African countries. We ensure that our news corpus does not include any duplicate by checking that no pair of articles contains either the same title or the same body.

### Seeds selection

We start from three manually selected seed key phrases describing the IPC phrases and that do not contain any common words: “food insecurity,” “hunger crisis,” and “famine.” We then consider as additional candidate seeds all the unigrams, bigrams, and trigrams mentioned in the 11.2 million articles. We convert each word of a candidate seed into an embedding vector such that words occurring in similar contexts end up close to one another in the embedding space (*17*). We then compute distances between seeds using the word mover’s distance, which measures the cost of traveling from one seed to another in the embedding space (*18*). The pretrained word embeddings and the implementation of the word mover’s distance were obtained from the Gensim package version 3.8.0 (https://radimrehurek.com/gensim/models/word2vec.html). We rank candidate seeds by their word mover’s distance to the 3 existing seeds and we keep the 100 candidate seeds closest to an existing seed, with bigrams and trigrams having to contain either the word “food” or the word “hunger” to be included.

### Frame-semantic parsing

Causal extraction refers to the natural language processing task of extracting cause-effect relations from text, which is distinct from the statistical analysis of cause and effect in the social sciences. We use a frame-semantic parser to extract semantic causes of food insecurity (*16*). The parser first splits each sentence into syntactic constituents *c*_1_, *c*_2_, …, *c_k_*, where each *c_i_* includes *p* ≥ 0 contiguous word tokens *w_j_*, *w*_*j* + 1_, …, *w*_*j* + *p*_ starting from position *j*. It then predicts a semantic role *t_i_* for each syntactic constituent *c_i_*. A semantic frame *f* is a collection of syntactic constituents along with their semantic roles (*c_i_*, *t_i_*)_*i* ∈ *f*_. To select text features associated with food insecurity, we adopt the following procedure:

1) First, we exclude semantic frames whose constituents’ roles do not include at least one “cause” and one “effect.” Note that there might be more than one “cause” and one “effect” per frame.
2) Next, we exclude semantic frames in which the “effect” constituents do not contain any seed key phrase related to food insecurity (fig. S3A).
3) Next, we exclude semantic frames that do not contain any of the causal links (fig. S3B) listed in the FrameNet lexical database version 1.7 (https://framenet.icsi.berkeley.edu/fndrupal/frameIndex).
4) Last, we select all the unigrams, bigrams, and trigrams mentioned either in a “cause” or in an “effect” constituent of the previously selected frames.

This procedure allows us to elicit 1062 text features from our corpus of news articles. To ensure that our text features also contain semantic causes of food insecurity established by experts, we handpick a list of 93 well-cited books and peer-reviewed studies on food insecurity from Google Scholar using the following search queries: “causes of famine,” “food insecurity causes,” “food insecurity Africa causes,” “causes of food crisis,” “famine Africa,” “famine Africa causes,” and “food crisis Africa causes.” We then run the parser on the text of these books and studies which reveals 149 additional text features (fig. S3D).

Overall, the frame-semantic parser allows us to extract 1211 text features semantically related to food insecurity. We choose a large set of 103 seeds as inputs to ensure that we do not miss important features. However, we find that the 13 most frequent seeds are sufficient for the frame-semantic parser to extract the same set of 1211 text features, and we therefore only focus on these 13 seeds in the rest of our analysis (fig. S3A). These 13 seeds contain the 3 initial seeds and 10 of the 100 candidate seeds. These 10 additional seeds are among the 29 candidates closest to the 3 initial seeds, which indicates that imposing a cutoff at 100 candidates has essentially no incidence on the results.

We used the version of the frame-semantic parser from 2 December 2018 (https://github.com/swabhs/open-sesame) (*15*). At the time of implementing our method, the parser was reaching state-of-the-art accuracy on the benchmark dataset FrameNet (*15*), and its performance is still very close to the current state of the art (*28*). One of the authors manually tested the parser on a random sample of 50 articles mentioning “food crisis” and established that it can correctly identify 89.74% of the causal frames.

### Keyword expansion

While the frame-semantic parser allows us to extract 1211 text features related to food insecurity, it fails to capture words semantically close to a seed that are also relevant. For example, the parser selects the word “terrorism” yet the word “terrorist,” which does not appear in any of the causal frames that we considered, is not picked up. For this reason, we expand our set of 1211 text features with semantically similar key phrases. We consider as candidate features all the unigrams in our news corpus and all the bigrams and trigrams occurring more than 1000 times. We compute the word mover’s distance between each original feature and each candidate feature, and we keep the candidates whose distance to an original feature is smaller than 6, obtaining 738 features.

### Dimensionality reduction

Having uncovered 1949 text features semantically related to food insecurity, we then discard the irrelevant ones using a Granger causality test (*29*). We define the following:

1) *y*_*d*, *t*_: IPC phase in district *d* during the quarter ending on month *t*.
2) *x*_*w*, *d*, *t*_: Proportion of articles mentioning text feature *w* and district *d* during month *t* (news factor).
3) *p*: Number of quarters of lags of the IPC phase.
4) *q*: Number of months of lags of the news factor.

We estimate a panel autoregressive distributed lag model of the IPC phase *y*_*d*, *t*_ in district *d* during the quarter ending on month *t*

$$y_{d,t}=a_{o}+a_{1}y_{d,t-3}+a_{2}y_{d,t-6}+\cdots +a_{p}y_{d,t-3p}+b_{1}x_{w,d,t-3}+\cdots +b_{q}x_{w,d,t-q-2}$$ (1)

where *p* > 0 and *q* > 0 are chosen on the basis of the Akaike information criterion. Each observation in Eq. 1 is at the district-quarter level. While news articles are time-stamped by the minute, they get aggregated into a district-month indicator by counting the number of articles mentioning a district and a key phrase in a month and dividing by the number of articles mentioning the district during the same month. Equation 1 is estimated using 4648 quarterly observations across 1162 districts over the period from July 2009 to April 2010 and evaluated using 1162 observations from July 2010. We reject the null hypothesis that *x_w_* does not Granger-cause *y* if the news factor and its lagged values whose coefficients are statistically different from zero add explanatory power to the regression according to an *F*-test at the 1% level. As explained in more detail below, news factors are selected over the period from July 2009 to July 2010 to ensure that no observation used for feature selection contributes to evaluating the predictive models’ performances.

Because the Granger causality test assumes stationarity, we take the first difference of each nonstationary news time series until it passes an augmented Dickey-Fuller test, a common statistical test used to determine whether a time series is stationary. We end up differencing 15 news factors—“slashed export,” “price rise,” “oppressive regimes,” “failed rains,” “migration,” “climate change,” “price of food,” “rising food prices,” “locusts,” “coup,” “severe rains,” “harvest decline,” “call for donations,” “cholera outbreak,” and “d’etat”—and we keep the differenced news indicators that Granger-cause the IPC phase for the rest of the analysis.

Of the 1949 text features previously selected, we retain the 167 that Granger-cause the IPC phase (fig. S3C). We find that expanding to candidate features up to a distance of 10 of an original feature does not lead to any additional relevant text features: the proportion of expansions passing the Granger causality test decreases up to a distance equal to 6 and becomes negligible thereafter (fig. S4).

### Categorizing text features

We start by manually partitioning the 167 text features into 12 semantically distinct clusters. We then convert each text feature into an indicator equal to one if it is mentioned in an article and zero otherwise, and we compute the pairwise correlations between text features on the entire corpus of news. Last, we ensure that the average correlation between a text feature and the other text features assigned to the same cluster is higher than with any other cluster (fig. S5).

### Traditional risk factors

We also collect traditional food insecurity risk indicators from recent studies on food insecurity (*8*, *12*, *31*). These risk indicators consist of the following:

1) a monthly count of violent conflict events and the monthly average number of fatalities per event,
2) a food prices index (monthly log nominal food price index and monthly year-on-year difference),
3) an evapotranspiration index (monthly mean) measuring the dissipation of water from plants and the soil into the atmosphere,
4) a rainfall index (monthly mean and deviation from average seasonal value),
5) a normalized difference vegetation index (monthly mean and deviation from average seasonal value),
6) a population count,
7) a terrain ruggedness index,
8) the district size,
9) the share of cropland use, and
10) the share of pasture use.

In other words, we collect district-month–level data on nine time-varying risk factors describing five different types of risk and district-level data on five time-invariant risk factors (fig. S6). The dataset covers 21 of the 37 countries in the FEWS NET dataset—Afghanistan, Burkina Faso, Chad, Democratic Republic of the Congo, Ethiopia, Guatemala, Haiti, Kenya, Malawi, Mali, Mauritania, Mozambique, Niger, Nigeria, Somalia, South Sudan, Sudan, Uganda, Republic of Yemen, Zambia, and Zimbabwe—over the period from July 2009 to July 2020.

### Random forest model

We define the following input variables as follows:

1) *p*: Province that district *d* belongs to.
2) *c*: Country that district *d* belongs to.
3) *v*_*k*,*d*,*t*_: Time-varying traditional factor *k* ∈ {1, …,9} in district *d* during month *t*
4) *v*_*k*,*p*,*t*_: Time-varying traditional factor *k* ∈ {1, …,9} in province *p* during month *t*.
5) *v*_*k*,*c*,*t*_: Time-varying traditional factor *k* ∈ {1, …,9} in country *c* during month *t*.
6) *v*_*l*,*d*_: Time-invariant traditional risk factor *l* ∈ {1, …,5} in district *d*.
7) *x*_*w*,*d*,*t*_: Proportion of articles mentioning text feature *w* ∈ {1, …,167} and district *d* during month *t*.
8) *x*_*w*,*p*,*t*_: Proportion of articles mentioning text feature *w* ∈ {1, …,167} and province *p* during month *t*.
9) *x*_*w*,*c*,*t*_: Proportion of articles mentioning text feature *w* ∈ {1, …,167} and country *c* during month *t*.
10) *y*_*d*,*t*_: IPC phase in district *d* during the quarter ending on month *t*.

To predict the IPC index using the traditional + news model, we estimate the following random forest (RF) regression

$$y_{d,t}=RF\left(\underset{n=1,\ldots ,6}{⋃}y_{d,t-3n};\underset{l=1,\ldots ,5}{⋃}v_{l,d};\underset{\begin{array}{rl} k & =1,\ldots ,9\\ i & \in \{d,p,c\}\\ n & =1,\ldots ,6 \end{array}}{⋃}v_{k,i,t-n-2};\underset{\begin{array}{rl} w & =1,\ldots ,167\\ i & \in \{d,p,c\}\\ n & =1,\ldots ,6 \end{array}}{⋃}x_{w,i,t-n-2}\right)$$ (2)

where *n* is in months. We omit the news factors *x*_*w*,*i*,*t*−*n*−2_ to estimate the traditional model. Similarly, we omit the traditional factors *v*_*l*, *d*_ and *v*_*k*,*i*,*t*−*n*−2_ to estimate the news model. The terms *v*_*k*,*p*,*t*_, *x*_*w*,*p*,*t*_,*v*_*k*,*c*,*t*_, and *x*_*w*,*c*,*t*_ account for shocks occurring in the province or the country that district *d* belongs to and that could affect the IPC phase in the district. The traditional model takes 167 input features: nine time-varying factors measured at the district, province, and country levels with 6 months of lagged values and five time-invariant indicators. By contrast, the news model takes 3006 additional input features: 167 news factors measured at the district, province, and country levels with 6 months of lagged values. All the models include six quarters of lagged values of the output variable.

Each observation in Eq. 2 is at the district-quarter level. The dataset used for the results presented in Fig. 3 contains 40,952 quarterly observations across 1162 districts in 21 countries over the period from July 2009 to February 2020. All the predictions only use information observed at least 3 months in the past. In other words, to predict the IPC phase next quarter, we incorporate monthly news indicators from the two previous quarters as separate independent variables. For example, the quarterly IPC phase for the 3-month period going from July to September is released in October. We predict its value using the six monthly indicators computed for each of the months between January and June.

All the country-specific results are produced with a single-panel regression model for all countries to have enough sample size. When comparing two models, we ensure that the exact same observations are available for both, and we skip the periods for which an observation is missing. The random forest regression is trained using scikit-learn version 1.1.1 (https://scikit-learn.org/stable/modules/generated/sklearn.ensemble.RandomForestRegressor.html). The hyperparameters are as follows:

1) n_estimators = {10, 20, 30, ..., 90, 100}
2) min_samples_split = {0.1, 0.5, 1, 2, 3, 4, 5}
3) max_features = {auto, sqrt, log_2_}
4) min_impurity_decrease = {10^-5^, 10^-4^, 10^-3^, 10^-2^, 10^-1^}

### Cross-validation

We estimate each random forest regression in the following way (fig. S7):

1) We temporally split the observation period into 10 folds.
2) Each fold is temporally split into training, validation, and test periods.
3) We train the model on the training period of each fold.
4) We evaluate the RMSE on the validation period for each combination of hyperparameters.
5) We find the hyperparameters that minimize the RMSE on the validation period.
6) We compute the RMSE on the test period using the optimal hyperparameters.
7) We report the unweighted average RMSE across the test periods of the 10 folds.

This standard temporal cross-validation procedure ensures that observations used for training a model occur before those of the corresponding test period. News indicators are selected by running Granger causality tests on the training and validation period of fold 1, which precedes all the test periods. We therefore guarantee that no observation used to select the news indicators contributes to evaluating the model’s performance. This procedure also implies that the optimal regularization parameters differ across each fold.

We tried increasing or burning training data by changing the duration of the first training fold by increments of 1 year. We found that, on average, each additional year of training data reduces the RMSE by 6 to 8% across models, which suggests that the current split does not favor one model versus another.

While the RMSE is typically used for continuous data, it is also suited to evaluate models of ordinal data (*31*). To test whether two forecasts are statistically different, we compare their mean squared errors computed by pulling together all the errors across test periods and using a Diebold-Mariano test with a 5% confidence level (*32*). We also derive a 95% confidence interval for the RMSE from the Diebold-Mariano statistics, which corrects for serial correlation across periods. We define the following:

1) *n*: Number of observations across test periods.
2) *e_i_*: Forecast error on observation *i*.
3) $\bar{e^{2}}$: Mean squared error.
4) γ*_k_*: Autocovariance at lag *k*.
5) $\sigma_{\bar{e^{2}}}$: SEM squared error.
6) CI: 95% confidence interval of the RMSE $\sqrt{\bar{e^{2}}}$.

$$\gamma_{k}=\frac{1}{n}\sum_{i=k+1}^{n}(e_{i}^{2}-\bar{e^{2}})(e_{i-k}^{2}-\bar{e^{2}})$$

$$\sigma_{\bar{e^{2}}}=\sqrt{\frac{\left[\gamma_{0}+2\;\sum_{k=1}^{\left(\;n^{1/3}\right)+1}\gamma_{k}\right]}{n}\;}$$

$$\mathrm{CI}=\left[\sqrt{\bar{e^{2}}-1.96\times \sigma_{\bar{e^{2}}}},\sqrt{\bar{e^{2}}+1.96\times \sigma_{\bar{e^{2}}}}\right]$$

### Classification of food crisis outbreaks

We define a food crisis outbreak as the date when the IPC phase raises to a value of 3 or more for at least two consecutive periods while the previous period’s phase is smaller or equal to 2. Let ${\hat{y}}_{m,d,t}$ be the IPC phase predicted by model *m* in district *d* during the quarter ending on month *t*, where *m*∈ {traditional, news, traditional + news}. We convert each previously estimated model of the IPC phase ${\hat{y}}_{m,d,t}$ into a classifier of food crisis outbreaks by introducing a lower threshold *l* and an upper threshold *u.* An outbreak is predicted to occur in district *d* during the quarter ending on month *t* if and only if

$${\hat{\mathrm{Outbreak}}}_{m,d,t}⟺\{\begin{array}{c} {\hat{y}}_{m,d,t+3}\;\geq u\\ {\hat{y}}_{m,d,t}\;\geq u\\ {\hat{y}}_{m,d,t-3}\;\leq l \end{array}$$

A model’s thresholds *l* and *u* determine its precision and recall. We vary *l* and *u* from 1 to 5 in increments of size 0.001 to estimate the model’s Pareto front, and we smooth it using a cuebiq spline interpolation (Fig. 3B). We report the AUC of each model and the recall obtained on the Pareto front for an 80% precision (Fig. 3C). The corresponding threshold values (*l*, *u*) are equal to (2.236, 3.125), (1.907, 2.712), and (2.105, 3.314) for the traditional, news, and traditional + news model, respectively.
