# Supplementary material for: Predicting food crises using news streams
Source: Sci Adv. 2023 Mar 3;9(9):eabm3449. doi: 10.1126/sciadv.abm3449 (PMC9984173; doi:10.1126/sciadv.abm3449)
Supplement: Supplementary file 1 — Supplementary Text Figs. S1 to S11 [file sciadv.abm3449_sm.pdf]

Science Advances
AAAS

Supplementary Materials for

Predicting food crises using news streams

Ananth Balashankar *et al.*

Corresponding author: Samuel P. Fraiberger, sfraiberger@worldbank.org

Sci. Adv. **9**, eabm3449 (2023)
DOI: 10.1126/sciadv.abm3449

This PDF file includes:

Supplementary Text
Figs. S1 to S11

S1 Robustness checks

In this section, we present some robustness checks showing that news factors consistently improve the predictions of food insecurity for a wide range of specifications.

S1.1 Alternative model specifications

Our preferred specification is the random forest regression model described in equation (2). Z-scoring all the input variables has no significant impact of the predictions according to a Diebold-Mariano test (p-value = 0.0619). We also tried estimating an OLS and a Lasso regression instead of the random forest (Fig S10A). Compared to a RMSE of 0.0819 obtained for random forest regression with traditional+news factors, a Lasso regression leads to a significantly higher RMSE of 10.002 (p-value = 0.0373) with 1,949 news factors, and of 10.012 with 167 news factors (p-value = 0.0455). An OLS regression with 167 factors also leads to a significantly higher RMSE of 0.0912 (p-value = 0.0483).

S1.2 Alternative spatial correlation assumptions

In equation (2) we introduce province- and country-level aggregate terms $v_{k,p,t}$, $x_{w,p,t}$, $v_{k,c,t}$, and $x_{w,c,t}$. We find that removing these terms leads to a significant deterioration of the predictions (Fig S10A) – e.g., the RMSE of the traditional+news model increases to 0.0921 (p-value = 0.0447).

We also tried incorporating spatial averages to account for the tendency of food insecurity to be spatially correlated. Let $\tilde{y}_{d,.}$ be the spatial average of $y_{d,.}$ computed using the 4 nearest neighbors of district d. $\tilde{x}_{.,d,.}$, $\tilde{v}_{.,d,.}$, $\tilde{v}_{.,d}$, $\tilde{x}_{.,p,.}$, $\tilde{v}_{.,p,.}$, $\tilde{v}_{.,p}$ $\tilde{x}_{.,c,.}$, $\tilde{v}_{.,c,.}$, and $\tilde{v}_{.,c}$ are defined in a similar fashion. We re-estimate equation (2) by including the following additional terms:

$$\left\{ \bigcup_{n=1,\dots,6} \tilde{y}_{d,t-3n} \bigcup_{l=1,\dots,5} \tilde{v}_{l,d} \bigcup_{\substack{k=1,\dots,9 \\ i\in\{d,p,c\} \\ n=1,\dots,6}} \tilde{v}_{k,i,t-n-2} \bigcup_{\substack{w=1,\dots,167 \\ i\in\{d,p,c\} \\ n=1,\dots,6}} \tilde{x}_{w,i,t-n-2} \right\}.$$

We find that is leads to an insignificant difference in RMSE – e.g., p-value = 0.1026 compared to the traditional+news model.

S1.3 Alternative text features

The 167 news factors included in the models presented in Fig. 3 are selected using a procedure which includes four steps: (i) choosing seed keyphrases related to food insecurity, (ii) expanding to semantic causes of food insecurity via frame-semantic parsing, (iii) expanding to semantically similar keyphrases using word embeddings, and (iv) removing non-predictive keyphrases through Granger causality. As demonstrated in the main text, this procedure uncovers features that are interpretable and validated by traditional indicators of food insecurity. To further support our approach, we demonstrate the contribution of each of these steps to the predictive performance of the traditional+news model (Fig. S10B). Compared to a RMSE of 0.819, we find that:

- Only including the 13 seed keyphrases with Porter stemming as features into the traditional model leads to a RMSE of 0.1404 (Diebold Mariano test, p-value = 0.0001). On the contrary, including the 13 seed keyphrases into the traditional+news model does not significantly change its predictions.
- Removing the parsing of news articles leads to a RMSE of 0.1271 (Diebold Mariano test, p-value = 0.002). For example, "collapse of government" would not have been picked up had this step been dropped.

- Removing the parsing of the 93 books and journal articles leads to a RMSE of 0.1077 (Diebold Mariano test, p-value = 0.0133). For example, "greenhouse gases" would not have been picked up had this step been dropped.
- Removing the keyword expansion leads to a RMSE of 0.1192 (Diebold Mariano test, p-value = 0.0087).
- Removing the dimensionality reduction with Granger causality leads to a RMSE of 0.1246 (Diebold Mariano test, p-value = 0.0074).
- Including all 1,949 text features in model (2) and using elastic net or XGBoost leads to RMSEs respectively equal to 0.0963 (Diebold Mariano test, p-value = 0.0120) or 0.1323 (Diebold Mariano test, p-value = 0.0008).

Taken together, these ablation studies indicate that all the steps of our method to discover relevant keyphrases are necessary to obtain large reductions in RMSE.

S1.4 Geolocating the news

Each news indicator is constructed by counting the cooccurrences of a text feature and geographic mentions. However, naive string matching of country, province, or district names could lead to false positives. For example, an article could be mentioning the text feature "conflict" and the country "Nigeria" even if no conflict is happening in Nigeria. To reduce the chance of false positives, we try a more conservative approach in which we only considered geographic units mentioned in the same sentence as a text feature. While the conservative approach is expected to reduce false positives, it could lead to more false negatives when, for example, the true location of an event is mentioned in a neighboring sentence. In practice, the conservative approach slightly increases the RMSE of the traditional+news model to 0.0928 (Diebold Mariano test, p-value =

0.0351), which suggests that occasional misclassifications of the locations where an event is occurring do not have much incidence on the results (Fig. S10B).

S1.5 Intensity of reporting

Measuring the proportion of news articles mentioning a text feature allows us to account for the intensity of reporting relative to the overall coverage that a district is receiving. In some cases, there could be a bias towards underreporting events, for example when an authoritarian regime controls the media, or overreporting events which are more headline-grabbing. As a robustness check, we try replacing each news indicator with a binary indicator equal to one if at least one article mentions a text feature in a month and zero otherwise. It degrades the RMSE of the traditional+news model to 0.1043 (Diebold Mariano test, p-value = 0.0261), which confirms that considering the multiplicity of articles mentioning a text feature is warranted (Fig. S10B).

Finally, we also tested whether the volume of news is predictive of food insecurity (Fig. S10B). We observe large variations in the volume of news across districts and over time, which prompted us to construct our news factors by counting the number of articles containing a text feature and normalizing by the volume of news within each district. In theory, one could assume news coverage going up or down as a crisis unfolds depending on the context. In practice, we find that including a time series measuring the district-level volume of news articles and 6 months of lagged values into the model did not significantly change the RMSE (Diebold Mariano test, p-value = 0.0785).

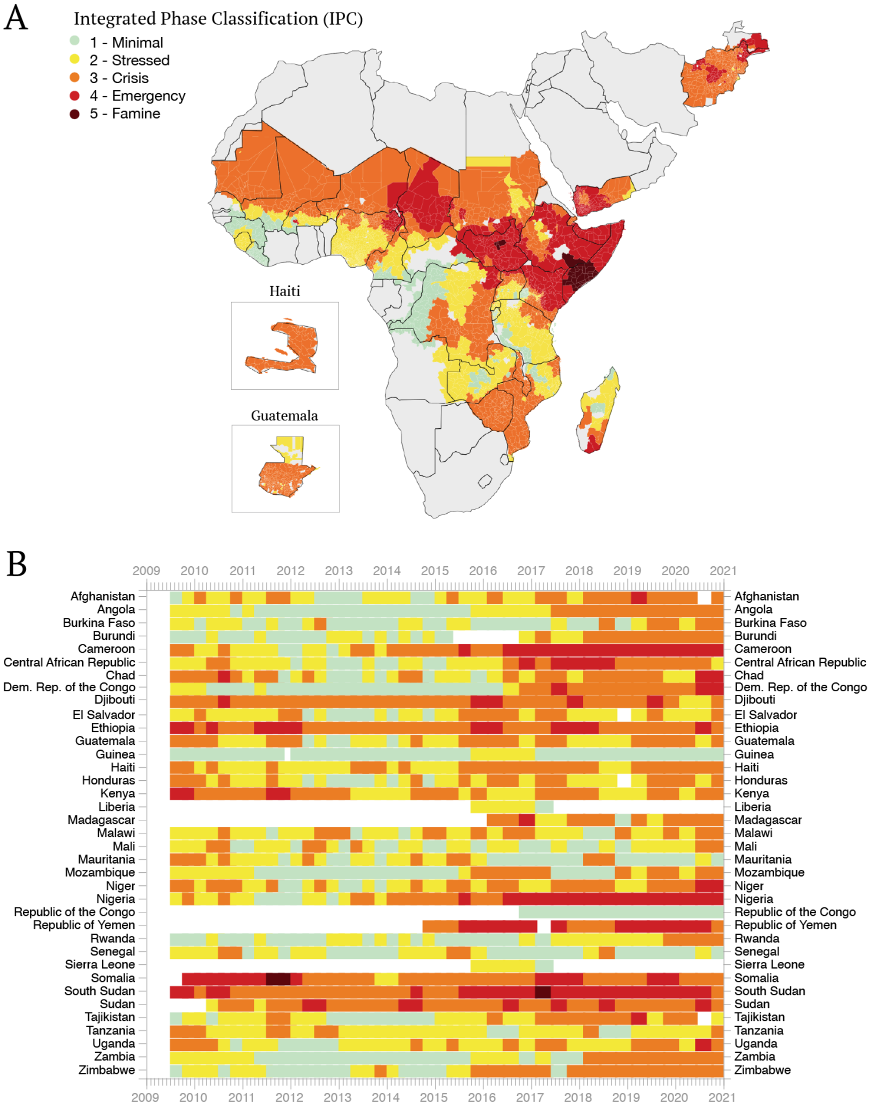

Fig. S1. **Food insecurity dataset.** (A) Integrated Phase Classification (IPC) of food security into 5 phases – minimal, stressed, crisis, emergency, and famine – at the district level across the 37 countries covered by the FEWS NET dataset. Each administrative unit is colored according to its maximum IPC phase over the period 2009-2020, revealing that food insecurity is geographically clustered. (B) Heatmap showing the maximum value of the IPC phase at the country level during each measurement period.

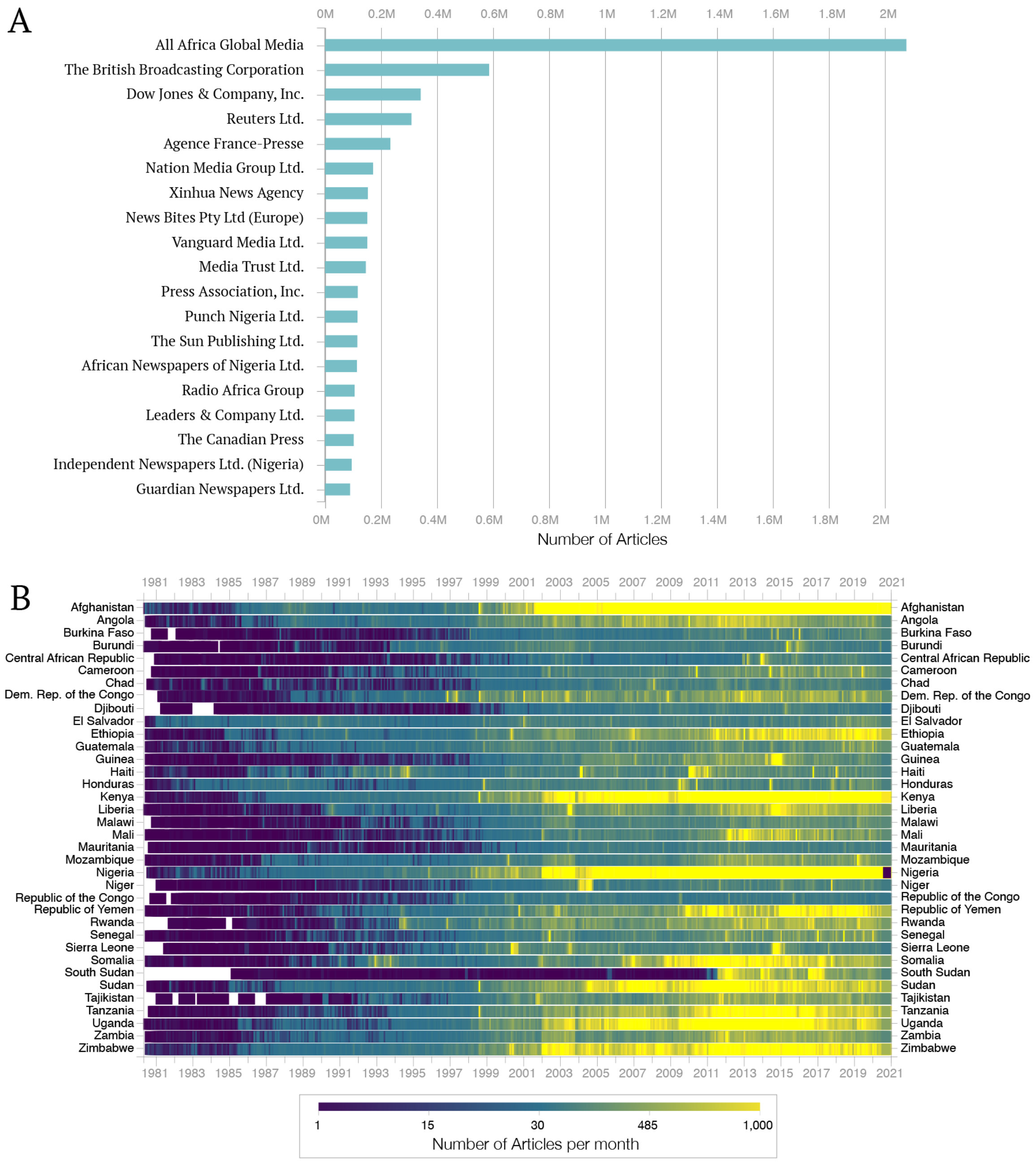

Fig. S2. News articles dataset. (A) The number of news articles grouped by publisher. (B) The number of news articles grouped by month and by country. We use the classification provided by Factiva to establish that an article focuses on a specific country.

A

Seed keyphrase	Number of articles containing frames
famine	25,637
food insecurity	25,404
malnourished	12,102
malnutrition	10,372
food crisis	10,154
starvation	8,012
hunger crises	6,518
shortage of food	5,482
life-threatening hunger	1,895
lack of food	1,266
scarcity of food	1,058
acute hunger	1,043
dearth of food	891

B

Causal link	Number of articles containing frames
since.c	10,548
see.v	10,142
result.n	9,016
cause.v	8,921
result (in).v	8,731
send.v	8,542
so.c	8,482
resulting.a	8,402
due to.prep	8,231
give rise.v	8,099
lead (to).v	7,601
leave.v	7,011
because.c	6,749
because of.prep	5,932
bring about.v	4,207
ravage.v	4,205
suffer.v	3,621
mean.v	3,047
bring.v	3,037
for.c	2,891
raise.v	2,462
render.v	2,145
bring on.v	2,088
force.v	1,904
reason.n	1,855
wreak.v	1,855
responsible.a	1,521
put.v	1,494
make.v	1,076
precipitate.v	1,047
cause.n	1,043
causative.a	980
sway.v	932
consequence.n	732
consequent.a	630
motivate.v	216
induce.v	108
dictate.v	107
resultant.a	67
consequential.a	29
legacy.n	0

C

Text feature	Number of articles
rise	1,377,032
conflict	754,194
coup	527,508
terrorism	519,898
corruption	515,890
flee	286,920
refugees	282,894
displaced	253,773
migration	220,003
drought	139,740
tragedy	106,500
climate change	89,612
siege	87,720
looting	71,207
floods	66,570
foreign troops	65,166
pirates	58,269
human rights abuses	57,870
economic crisis	53,800
catastrophe	50,612
repression	47,806
foreign aid	42,924
secession	42,924
land reform	41,067
price rise	39,474
natural disaster	36,672
the offensive	34,698
humanitarian situation	34,453
blockade	34,132
food insecurity	31,608
power struggle	29,977
asylum seekers	25,558
overthrow	23,220
food crisis	23,135
malnourished	23,134
cyclone	22,822
apathy	22,556
farmland	22,355
military junta	21,493
air attack	19,920
military dictatorship	19,916
bombing campaign	19,563
dysfunction	19,332
food assistance	18,324
militia groups	18,119
dehydrated	17,688
epidemics	17,647
dictators	17,620
convoys	17,219
cholera outbreak	17,156
corrupt government	16,853
environmental degradation	16,565
infant mortality	16,418
humanitarian disaster	15,271
totalitarian	14,926
carbon	14,456
civil strife	13,991
pests	13,884
international intervention	12,933
land grab	12,849
gastrointestinal	12,736
d'etat	12,597
rising inflation	11,059
police torture	10,523
jihadist groups	10,231
land seizures	10,151
slave trade	10,112
international terrorists	10,059
warlord	10,045
rebel insurgency	10,030
burning houses	9,967
ecological crisis	9,965
price of food	9,861
terrorist	9,824
politically engineered	9,713
reduced national output	9,562
lack of rains	9,526
internal strife	9,523
years of warfare	9,517
self reliance	9,473
major offensive	9,440
alarming level	9,421
water availability	9,294
transport bottleneck	9,160
inadequate rainfall	9,140

hunger crises	9,114
aid appeal	9,099
rinderpest	9,047
land invasions	9,037
brutal government	8,831
without international aid	8,760
collapse of government	8,753
massive starvation	8,588
locusts	8,566
mass hunger	8,541
greenhouse gases	8,527
mayhem	8,504
rising food prices	7,913
call for donations	7,840
withheld relief	7,748
brain drain	7,744
potato blight	7,733
clans	7,491
gangs of bandits	7,490
water distribution shortages	7,384
lack of authority	7,257
continued strife	7,199
infrastructure damage	7,039
harvest decline	6,871
pushing peasants off	6,712
mismanagement	6,645
scanty rainfall	6,580
forests destroyed	6,576
toll on livestock	6,523
makeshift camps	6,457
cycle of poverty	6,435
wreaked havoc	6,419
failed crops	6,381
harvests are devastated	6,337
bad harvests	6,205
stolen food aid	6,058
destructive pattern	5,862
slashed export	5,801
regimes were toppled	5,783
land degradation	5,770
increased external debt	5,717
man-made disaster	5,688
prolonged fighting	5,625
cattle death	5,608
disrupted trade	5,578
prolonged dry spell	5,545
rival warlords	5,373
authoritarian	5,106
shortage of rains	4,801
economic impoverishment	4,510
severe rains	4,435
failed rains	4,429
life-threatening hunger	4,314
violent suppression	4,155
international embargo	4,134
disruption to farming	3,690
restricted humanitarian access	3,627
clan warfare	3,623
collapsing economy	3,459
climatic hazards	3,446
weather extremes	3,435
poor soil quality	3,310
acute hunger	2,997
lack of agricultural infrastructure	2,877
lack of roads	2,772
reduced imports	2,768
population crisis	2,765
cattle plague	2,643
continued deterioration	2,593
clan battle	2,370
aid workers died	2,154
international alarm	2,102
devastated the economy	1,853
anti-western policies	1,837
lack of alternatives	1,768
livestock had died	1,668
abnormally low rainfall	1,626
oppressive regimes	1,564
lack of cultivation	1,551
restricted relief flights	1,363
unable to sow	1,136
civilians uprooted	1,087

D

Author & title

" Brinkman, CS Hendrix",Food Insecurity and Violent Conflict: Causes
" Clay, BK Holcomb",Politics and the Ethiopian famine: 1984-1985
" Ilaboya, E Atikpo, FE Omofuma…","Causes, effects and way forward to food insecurity"
" Ilaboya, E Atikpo, FE Omofuma…","Causes, effects and way forward to food insecurity"
" Legwegoh, EDG Fraser",Food crisis or chronic poverty: metanarratives of food insecurity in Sub-Saharan Africa
" Smith, AE El Obeid, HH Jensen",The geography and causes of food insecurity in developing countries
"Borji, M Moradi, M Otaghi…","Relationship between nutritional status, food insecurity, and causes of hospitalization of children with infectious diseases"
"Clapp, MJ Cohen",The global food crisis: Governance challenges and opportunities
"Eneyew, W Bekele",Causes of household food insecurity in Wolayta: Southern Ethiopia
"Headey, S Fan",Reflections on the global food crisis: How did it happen? How has it hurt? And how can we prevent the next one?
"Hesselberg, JA Yaro",An assessment of the extent and causes of food insecurity in northern Ghana using a livelihood vulnerability framework
"Kidane, ZG Alemu, G Kundhlande","Causes of household food insecurity in Koredegaga peasant association, Oromiya zone, Ethiopia"
"Meng, N Qian, P Yared","The institutional causes of China's Great Famine, 1959–1961"
"Messer, MJ Cohen","Conflict, food insecurity and globalization"
"Mokyr, CÓ Gráda",What do people die of during famines: the Great Irish Famine in comparative perspective
"Musemwa, V Muchenje, A Mushunje, F Aghdasi…","Household food insecurity in the poorest province of South Africa: level, causes and coping strategies"
"Okori, J Obua, V Baryamureeba",Famine disaster causes and management based on local community's perception in Northern Uganda
"Owusu, F Ng'ambi",Structural damage: The causes and consequences of Malawi's food crisis
"Von Braun, T Teklu, P Webb","Famine in Africa: Causes, responses, and prevention"
Abdalla,Causes of food insecurity in Southern Africa: An assessment
Alex De Waal et al., New Variant Famine: Aids and Food Crisis in Southern Africa.
Alex De Waal, War and Famine in Africa
Alinovi et al., Measuring household resilience to food insecurity: application to Palestinian households
Amartya Sen, Poverty and Famines..an Essay on Entitlement..and Deprivation.
Babatunde, Determinants of vulnerability to food insecurity: A genderbased analysis of farming households in Nigeria
Ball,Understanding the causes of African famine
Barrett, Measuring food insecurity
Bastiaan T. Heijmansa Et Al, Persistent Epigenetic Differences Associated With.prenatal Exposure to Famine in Humans.
Battisti et al., Historical warnings of future food insecurity with unprecedented seasonal heat
Borch, Food security and food insecurity in Europe: An analysis of the academic discourse (1975–2013)
Bowbrick,Rejoinder: an untenable hypothesis on the causes of famine
Bowbrick,The causes of famine: a refutation of Professor Sen's theory
Brookings Institute, Somalia: Drought + Conflict = Famine?
Bryceson, Food insecurity and the social division of labour in Tanzania, 1919-85
Campbell, Food insecurity: a nutritional outcome or a predictor variable?
Chand,"The global food crisis: causes, severity and outlook"
Daniel Maxwell et al., The 2011 Somalia Famine: Context, Causes, and Complications
Deng, Famine in the Sudan: causes, preparedness and response: a political, social and economic analysis of the 1998 Bahr el Ghazal
Devereux, Food insecurity in Ethiopia
Donald Curtis et al., Preventing Famine.policies and Prospects.for Africa.
Eneyew et al., Causes of household food insecurity in Wolayta: Southern Ethiopia
Fawole, E Ilbasmis, B Ozkan","Food insecurity in Africa in terms of causes, effects and solutions: A case study of Nigeria
Foley, An ecological analysis of factors associated with food insecurity in South Australia, 2002–7
Gebre-egziabher Kirosa et al., War, Famine and Excess Child Mortality.in Africa: the Role of Parental Education.
Golay,The food crisis and food security: Towards a new world food order?
Gonzalez,"The global food crisis: law, policy, and the elusive quest for justice"
Hart, Exploring definitions of food insecurity and vulnerability: time to refocus assessments
Hendriks, The food security continuum: a novel tool for understanding food insecurity as a range of experiences
Hendrix, Food insecurity and conflict dynamics: Causal linkages and complex feedbacks
Humanitarian Practice Network, Humanitarian Exchange
Jaeger,The causes of Africa's food crisis
Jaeger,The causes of Africa's food crisis
Jane Corbett, Famine and Household Coping Strategies
Jeffrey Clark, Famine in Somalia and The.international Response:.collective Failure
Jo Allen Fair., The Body Politic, the Bodies of Women, and the Politics of Famine in U.s. Television Coverage of Famine in the Horn of Africa,
John Seaman, Famine Mortality in Africa
John Wagner Givens, An Analysis Of..frank Dikötter's.mao's Great Famine.
Joseph C. Miller, The Significance of Drought, Disease And.famine in the Agriculturally Marginal.zones of West-central Africa
K Darkoh,The underlying causes of the food crisis in Africa
K Darkoh,The underlying causes of the food crisis in Africa
Keen,The benefits of famine: a political economy of famine and relief in Southwestern Sudan 1983-89
Lisa Cliggett, Grains from Grass: Aging, Gender and Famine in Rural Africa
Mamadou Baro et al., Persistent Hunger:.perspectives On.vulnerability, Famine,.and Food Security In.sub-saharan Africa
Mark Duffield, War and Famine in Africa
McPherson,The global food crisis: causes and solutions
Mellor, S Gavian","Famine: Causes, prevention, and relief
Menon,Famine in Malawi: Causes and consequences
Michael Watts, Entitlements or Empowerment Famine and Starvation in Africa.
Misselhorn,"Food Insecurity in Southern Africa: Causes and emerging response options from evidence at regional, provincial and local scales
Misselhorn,What drives food insecurity in southern Africa? A meta-analysis of household economy studies
Noah Zerbe, Feeding the Famine? American Food Aid and The.gmo Debate in Southern Africa
Olsson,"On the causes of famine: drought, desertification and market failure in the Sudan"
Otaha, Food insecurity in Nigeria: Way forward
Perez-Escamilla, Food insecurity measurement and indicators
Peter Singer, Famine, Affluence, and Morality.
Ramakrishna, An empirical analysis of food insecurity in Ethiopia: the case of North Wello
Rosset,Food sovereignty and the contemporary food crisis
Rubey,Malawi's food crisis: Causes and solutions
Sen,The causes of famine: a reply
Siamwalla et al, Food insecurity in developing countries
Singh,"Global food crisis: magnitude, causes and policy measures"
Stephan Haggard et al., Hunger And.human Rights:.the Politics of Famine.in North Korea
Stephen Devereux, Famine in the Twentieth Century
Stephen Devereux., Why Does Famine Persist in Africa?
Swift,Understanding and preventing famine and famine mortality
Undp Somalia, Somalia Drought.impact & Needs.assessment.volume I..synthesis Report.
Walker,Famine in Africa—the real causes and possible solutions
Webb, Addressing the" In" in food insecurity
Weiser, Conceptual framework for understanding the bidirectional links between food insecurity and HIV/AIDS
Welderufael, Determinants of households vulnerability to food insecurity in Ethiopia: econometric analysis of rural and urban households

Fig. S3. Frame-semantic parsing. (A) The 13 target keywords used to select semantic frames related to food insecurity along with the number of news articles in which the selected frames appear. To account for possible inflections, we use the Porter stemming algorithm on each word token and we select from our news corpus semantic frames matching the root words. (B) The 41 causal links obtained from the FrameNet lexical database used to select relevant semantic frames, along with the number of news articles in which the selected frames appear. (C) The 167 text features used in our predictive model along with the number of news articles in which they appear. (D) The 93 books and peer-reviewed studies on which we run the frame-semantic parser.

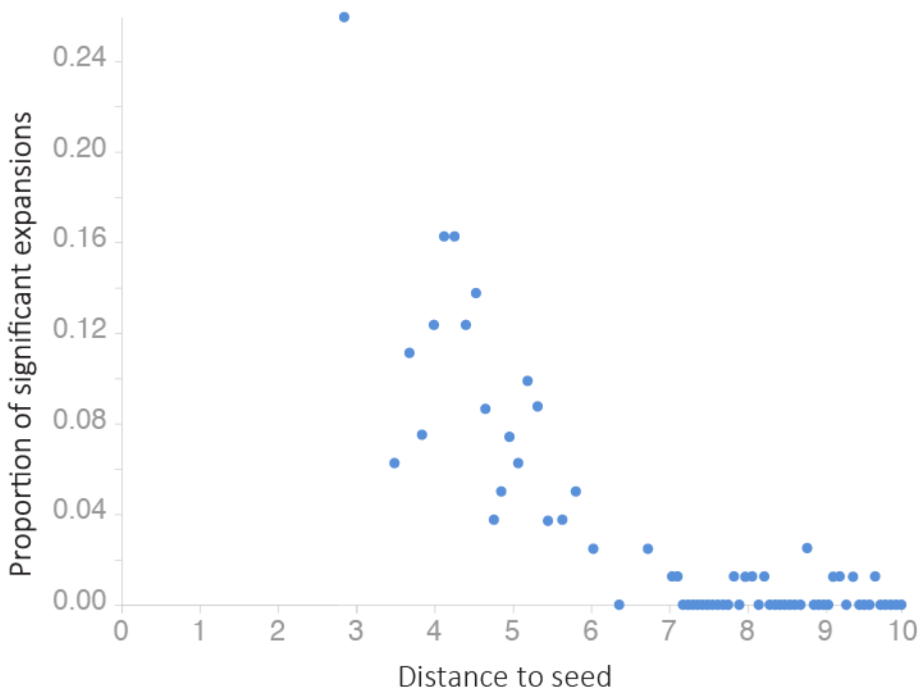

Fig. S4. Keyword expansion. Starting from the 1,211 original features obtained by frame-semantic parsing, we find 5,228 candidate features mentioned in the news and with a word mover's distance to an original feature smaller than 10. After ranking candidate features by increasing distance to a seed and partitioning them into 50 groups of equal size, we report the proportion of candidate features within each group passing the Granger causality test (y-axis) and the average distance to an original feature within each group (x-axis). As the distance to a seed gets close to 6, the proportion of candidate features predicting the IPC phase approaches zero, providing support to our choice of exploring the space of semantic neighbors up to a distance of 6.

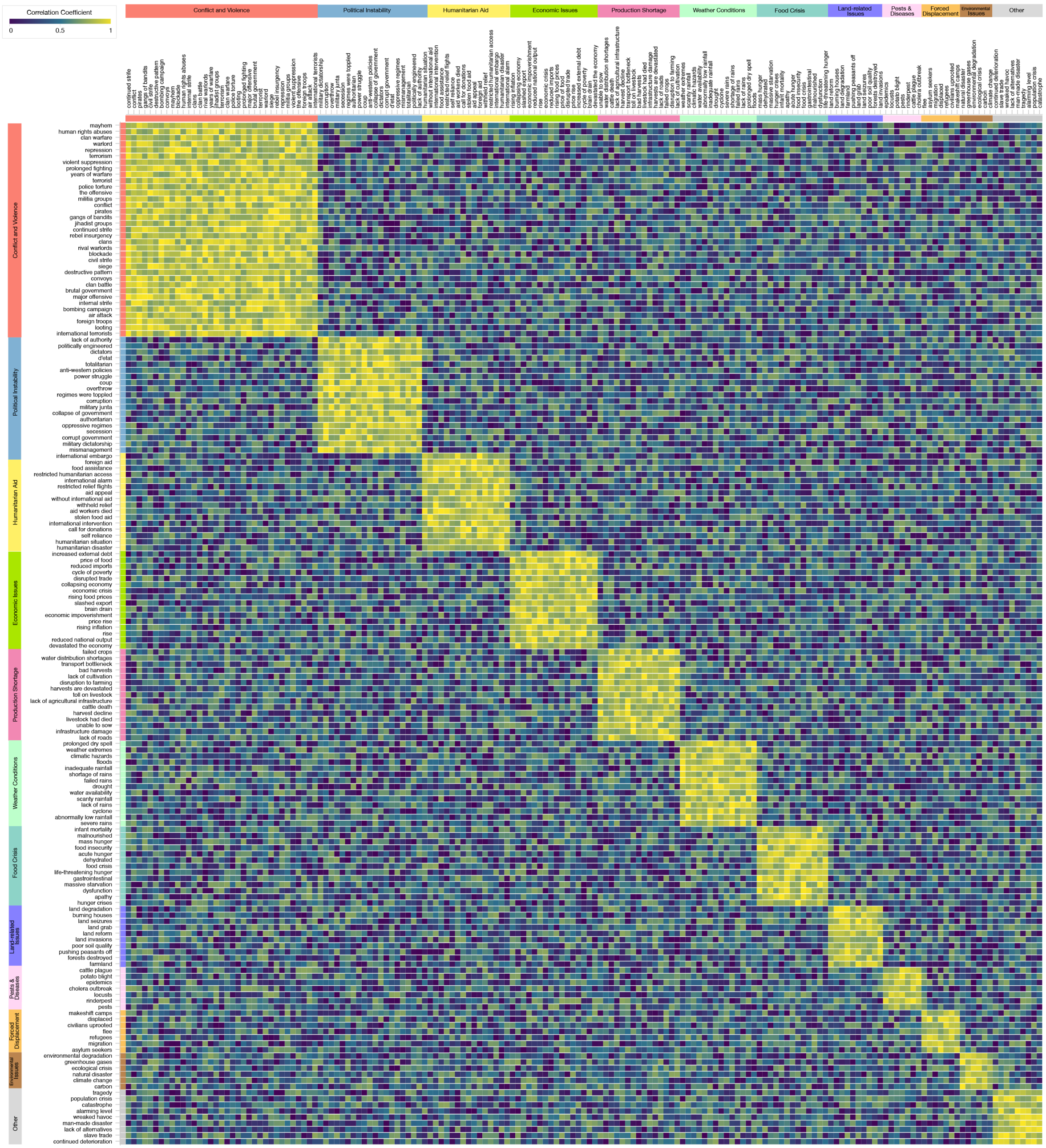

Fig. S5. Categorizing text features. Pairwise correlation between news factors over the period June 1980 - July 2020, showing an average correlation between news factors in the same cluster about twice as high as that of factors belonging to different clusters (69.9% versus 34.9%), which provides support to our categorization.

A

Percentage of news articles mentioning feature	mean	std	min	25%	50%	75%	max
rise	30.27	23.47	0	19.63	31.75	48.09	87.05
coup	22.97	14.53	0	15.34	23.63	33.93	67.97
corruption	19.69	14.37	0	12.75	20.59	30.42	60.18
terrorism	18.89	14.42	0	12.01	19.43	29.62	56.06
conflict	17.21	8.68	0	12.99	17.62	23.70	39.68
refugees	17.18	10.64	0	11.66	17.75	25.01	50.11
flee	15.49	10.71	0	10.06	16.22	23.54	50.34
displaced	15.38	10.08	0	10.36	16.12	22.92	47.12
drought	14.46	7.48	0	10.73	14.93	20.15	36.01
climate change	11.92	5.99	0	9.31	12.78	16.69	35.87
migration	10.73	9.38	0	6.21	11.06	17.66	35.85
tragedy	9.20	6.53	0	6.01	9.61	14.13	28.36
siege	8.79	5.92	0	5.71	9.22	13.30	28.28
economic crisis	8.68	4.64	0	6.61	8.99	12.17	22.52
looting	8.65	5.34	0	5.9	9.05	12.71	25.88
pirates	8.57	4.83	0	6	8.98	12.25	23.91
foreign troops	8.47	5.11	0	5.73	8.73	12.25	23.42
floods	6.81	5.16	0	4.25	7.23	10.85	23.10
foreign aid	6.55	4.14	0	4.55	6.80	9.66	18.20
human rights abuses	6.42	4.81	0	4.03	6.63	10.02	20.45
malnourished	6.28	3.04	0	4.7	6.56	8.60	16.97
secession	6.27	4.14	0	4.09	6.52	9.40	19.67
dehydrated	5.67	2.66	0	4.29	5.88	7.70	14.53
convoys	5.58	2.62	0	4.18	5.76	7.55	14.68
price rise	5.46	3.97	0	3.55	5.64	8.44	15.24
repression	5.45	4.37	0	3.26	5.60	8.72	17.73
humanitarian situation	5.34	3.71	0	3.63	5.52	8.08	15.43
land reform	5.19	4.05	0	3.26	5.36	8.24	16.22
the offensive	5.17	3.73	0	3.39	5.43	7.97	16.09
catastrophe	5.16	4.50	0	3.06	5.34	8.50	17.32
blockade	5.01	3.70	0	3.15	5.28	7.79	16.66
overthrow	4.75	3.05	0	3.17	5.01	7.07	14.20
cyclone	4.69	3.02	0	3.15	4.93	7.02	14.62
military junta	4.69	2.93	0	3.22	4.87	6.90	13.31
pests	4.68	2.36	0	3.47	4.89	6.47	12.51
asylum seekers	4.61	3.20	0	2.96	4.80	6.99	14.54
natural disaster	4.50	3.83	0	2.76	4.76	7.36	15.03
power struggle	4.40	3.46	0	2.71	4.51	7.00	14.07
gastrointestinal	4.33	2.26	0	3.16	4.53	6.04	11.44
military dictatorship	4.28	2.82	0	2.88	4.48	6.40	12.95
dysfunction	4.25	2.78	0	2.8	4.37	6.31	12.85
militia groups	4.14	2.69	0	2.72	4.28	6.16	12.74
d'etat	4.08	2.24	0	2.91	4.21	5.76	11.76
bombing campaign	4.04	2.80	0	2.63	4.25	6.19	12.59
apathy	4.01	3.00	0	2.42	4.15	6.24	13.12
warlord	3.98	2.00	0	2.93	4.15	5.50	10.87
cholera outbreak	3.93	2.62	0	2.63	4.12	5.92	11.48
air attack	3.93	2.82	0	2.51	4.10	6.06	11.25
farmland	3.91	2.99	0	2.4	4.12	6.19	13.06
food insecurity	3.88	3.56	0	2.18	4.06	6.58	13.84
dictators	3.79	2.65	0	2.42	3.92	5.76	12.32
corrupt government	3.71	2.60	0	2.39	3.81	5.66	10.95
food crisis	3.61	3.04	0	2.21	3.71	5.88	11.54
police torture	3.55	2.05	0	2.51	3.72	5.13	9.99
rinderpest	3.52	1.90	0	2.55	3.68	4.96	9.62
humanitarian disaster	3.44	2.47	0	2.26	3.58	5.29	10.36
epidemics	3.39	2.66	0	2.03	3.53	5.44	11.39
carbon	3.36	2.40	0	2.1	3.48	5.16	10.73
infant mortality	3.35	2.56	0	1.99	3.47	5.28	11.45
environmental degradation	3.29	2.57	0	2.09	3.44	5.25	9.68
civil strife	3.27	2.37	0	2.15	3.45	5.11	10.14
international intervention	3.26	2.27	0	2.21	3.36	4.99	9.04
rebel insurgency	3.19	2.00	0	2.15	3.33	4.72	9.65
locusts	3.17	1.85	0	2.21	3.26	4.55	9.41
food assistance	3.15	2.71	0	1.79	3.28	5.18	10.50
jihadist groups	3.10	2.02	0	2.01	3.21	4.62	9.15
clans	3.01	1.73	0	2.05	3.10	4.31	8.89
terrorist	2.98	1.98	0	2	3.07	4.48	8.46
burning houses	2.97	2.00	0	1.9	3.06	4.46	9.35
totalitarian	2.96	2.44	0	1.78	3.13	4.80	10.31
scanty rainfall	2.93	1.62	0	2.05	3.04	4.16	7.98
international terrorists	2.88	2.01	0	1.89	2.98	4.36	8.33
aid appeal	2.83	1.91	0	1.93	2.90	4.27	8.35
rising inflation	2.82	2.10	0	1.85	2.99	4.44	7.59
without international aid	2.76	1.87	0	1.83	2.83	4.16	8.02
makeshift camps	2.76	1.61	0	1.94	2.88	3.99	8.12
land grab	2.74	2.27	0	1.63	2.84	4.42	9.68
potato blight	2.72	1.76	0	1.85	2.85	4.05	8.33
internal strife	2.67	1.95	0	1.7	2.72	4.13	8.23
reduced national output	2.66	1.96	0	1.73	2.76	4.13	7.98
collapse of government	2.64	1.87	0	1.76	2.74	4.05	7.35
brutal government	2.61	1.88	0	1.67	2.68	4.02	7.76
greenhouse gases	2.61	1.85	0	1.68	2.75	4.02	8.40
slave trade	2.59	2.01	0	1.54	2.67	4.11	7.57

Percentage of news articles mentioning feature	mean	std	min	25%	50%	75%	max
ecological crisis	2.59	2.00	0	1.68	2.74	4.14	7.41
major offensive	2.57	1.94	0	1.64	2.68	4.03	7.88
mayhem	2.54	1.84	0	1.63	2.63	3.92	8.00
inadequate rainfall	2.51	1.91	0	1.56	2.66	3.95	8.52
self reliance	2.51	1.95	0	1.58	2.66	3.99	8.12
withheld relief	2.47	1.76	0	1.57	2.59	3.80	7.63
price of food	2.42	1.99	0	1.48	2.56	3.90	8.17
land seizures	2.42	2.02	0	1.42	2.54	3.93	8.63
alarming level	2.41	1.94	0	1.51	2.47	3.87	7.34
call for donations	2.39	1.77	0	1.52	2.48	3.72	7.64
politically engineered	2.39	1.97	0	1.42	2.46	3.86	8.40
years of warfare	2.39	1.95	0	1.49	2.53	3.92	7.74
lack of rains	2.38	1.95	0	1.48	2.46	3.83	7.40
brain drain	2.37	1.76	0	1.49	2.47	3.70	7.65
gangs of bandits	2.35	1.73	0	1.47	2.45	3.65	7.79
hunger crises	2.34	1.91	0	1.45	2.45	3.79	7.63
continued strife	2.32	1.70	0	1.56	2.44	3.63	6.72
cattle death	2.32	1.50	0	1.54	2.41	3.46	7.11
land invasions	2.30	1.90	0	1.36	2.42	3.76	8.15
forests destroyed	2.28	1.62	0	1.48	2.41	3.52	7.59
lack of authority	2.25	1.70	0	1.48	2.33	3.55	6.50
rising food prices	2.20	1.78	0	1.3	2.28	3.54	7.17
water availability	2.18	1.93	0	1.25	2.29	3.62	8.05
transport bottleneck	2.17	1.91	0	1.24	2.32	3.62	7.81
increased external debt	2.15	1.51	0	1.39	2.21	3.28	6.30
mass hunger	2.12	1.85	0	1.25	2.22	3.49	7.49
rival warlords	2.12	1.47	0	1.35	2.20	3.22	6.80
international embargo	2.09	1.29	0	1.45	2.15	3.05	5.78
mismanagement	2.09	1.63	0	1.27	2.20	3.31	6.85
economic impoverishment	2.05	1.34	0	1.44	2.13	3.07	4.97
pushing peasants off	2.05	1.64	0	1.25	2.12	3.28	6.82
slashed export	2.04	1.52	0	1.23	2.12	3.18	6.08
regimes were toppled	2.03	1.52	0	1.22	2.09	3.17	6.72
authoritarian	2.03	1.43	0	1.3	2.10	3.10	5.82
massive starvation	2.01	1.85	0	1.1	2.12	3.42	7.28
destructive pattern	1.97	1.53	0	1.25	2.07	3.13	6.25
cycle of poverty	1.97	1.60	0	1.22	2.06	3.16	6.22
water distribution shortages	1.96	1.72	0	1.12	2.07	3.26	6.86
infrastructure damage	1.95	1.68	0	1.12	2.02	3.21	6.56
stolen food aid	1.94	1.56	0	1.16	2.03	3.11	6.76
prolonged fighting	1.93	1.50	0	1.19	2.00	3.07	6.23
wreaked havoc	1.90	1.60	0	1.09	1.97	3.11	6.36
harvest decline	1.85	1.66	0	1.07	1.98	3.15	6.36
disrupted trade	1.85	1.49	0	1.15	1.96	2.99	5.78
toll on livestock	1.85	1.62	0	1.06	1.94	3.06	6.75
life-threatening hunger	1.83	1.31	0	1.18	1.91	2.81	5.77
failed crops	1.82	1.60	0	1.08	1.90	3.04	6.19
harvests are devastated	1.77	1.59	0	0.99	1.84	2.95	6.48
bad harvests	1.77	1.58	0	1.01	1.87	2.97	6.28
prolonged dry spell	1.71	1.49	0	1.01	1.82	2.84	5.46
failed rains	1.70	1.33	0	1.02	1.75	2.69	5.28
clan warfare	1.69	1.20	0	1.08	1.77	2.62	5.56
man-made disaster	1.68	1.51	0	0.95	1.75	2.81	6.07
land degradation	1.68	1.52	0	0.91	1.74	2.81	6.11
severe rains	1.67	1.33	0	0.98	1.74	2.68	5.77
collapsing economy	1.65	1.18	0	1.11	1.75	2.57	4.59
restricted humanitarian access	1.62	1.20	0	1.05	1.67	2.52	5.26
shortage of rains	1.59	1.39	0	0.9	1.65	2.64	5.59
cattle plague	1.59	1.03	0	1.05	1.64	2.36	4.99
weather extremes	1.50	1.17	0	0.94	1.56	2.38	4.73
poor soil quality	1.46	1.15	0	0.89	1.50	2.32	4.59
violent suppression	1.46	1.29	0	0.82	1.51	2.43	5.26
disruption to farming	1.44	1.21	0	0.87	1.52	2.37	4.61
population crisis	1.40	1.05	0	0.91	1.44	2.20	4.25
climatic hazards	1.39	1.17	0	0.84	1.49	2.27	4.86
clan battle	1.37	0.97	0	0.87	1.44	2.12	4.40
aid workers died	1.35	0.93	0	0.87	1.41	2.05	3.77
international alarm	1.34	0.92	0	0.89	1.39	2.02	3.74
lack of roads	1.31	1.05	0	0.81	1.35	2.10	4.30
lack of agricultural infrastructure	1.28	1.07	0	0.75	1.32	2.08	4.20
reduced imports	1.28	1.05	0	0.77	1.35	2.08	4.17
continued deterioration	1.28	1.02	0	0.79	1.30	2.03	3.87
civilians uprooted	1.26	0.66	0	0.9	1.29	1.76	3.53
acute hunger	1.25	1.09	0	0.72	1.33	2.09	4.58
livestock had died	1.20	0.82	0	0.75	1.23	1.81	3.76
anti-western policies	1.18	0.86	0	0.76	1.21	1.81	3.64
devastated the economy	1.14	0.86	0	0.74	1.20	1.79	3.43
lack of alternatives	1.07	0.84	0	0.68	1.11	1.70	3.28
abnormally low rainfall	1.01	0.81	0	0.61	1.05	1.61	3.39
restricted relief flights	0.99	0.74	0	0.62	1.02	1.55	3.17
lack of cultivation	0.94	0.79	0	0.57	0.99	1.54	3.21
oppressive regimes	0.84	0.79	0	0.43	0.87	1.43	3.22
unable to sow	0.78	0.67	0	0.45	0.82	1.29	2.64

B

	count	mean	std	min	25%	50%	75%	max
Population count	1,162	629,983.06	1,087,278.88	2,245.00	137,902.00	266,441.00	602,916.00	14,050,941.00
Terrain ruggedness	1,162	96,378.43	127,219.22	1,339.26	17,795.50	45,881.85	118,921.10	1,046,065.00
District size (in square kilometers)	1,162	14,282.75	28,097.42	10.30	2,198.55	5,834.55	13,339.02	331,292.00
Pasture area share	1,162	49.76	26.73	0.00	29.16	52.05	70.56	99.60
Cropland area share	1,162	29.97	25.92	0.00	7.38	24.27	45.70	99.24
Rainfall index	148,736	11.47	14.17	0.00	0.51	4.79	19.34	124.22
Evapotranspiration index	148,700	10.99	10.58	0.00	1.56	6.90	19.83	47.90
Conflict fatality count	148,736	3.00	25.82	0.00	0.00	0.00	0.00	2,394.00
Monthly log nominal food price index	148,736	2.58	7.53	0.34	1.01	1.23	1.73	140.00
Conflict event count	148,736	1.07	5.79	0.00	0.00	0.00	0.00	265.00
Vegetation index	148,736	0.40	0.21	-0.04	0.21	0.37	0.59	0.86

Fig. S6. Summary statistics. (A) Summary statistics of the proportion of news mentions of the 167 news factors across N = 643,986 district-month observations. (B) Summary statistics of the traditional factors obtained from previous research (*8*) across districts and months.

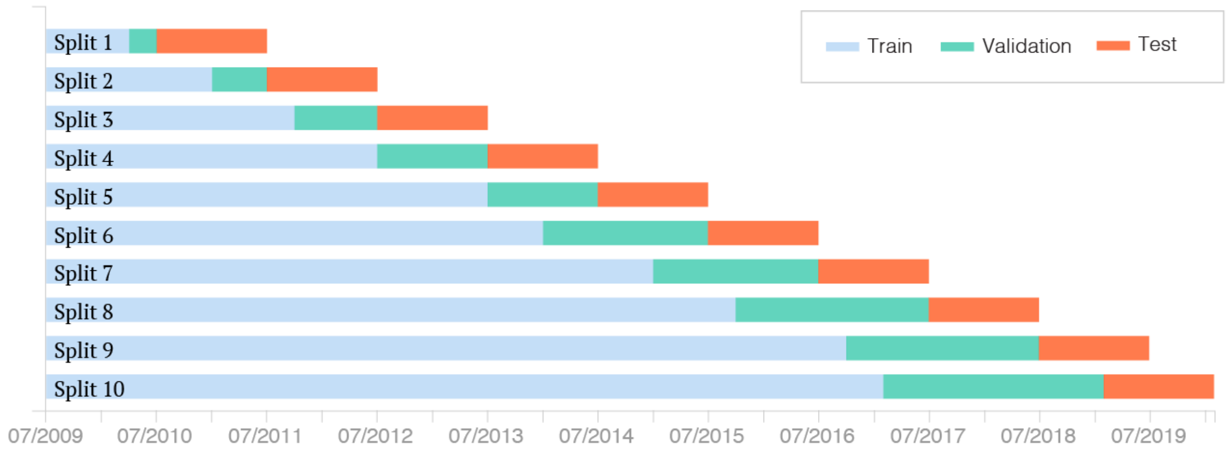

Fig. S7. Cross-validation. A timeline describing our cross-validation methodology. We temporally split the observation period into 10 folds. Each fold is temporally split into training, validation, and test periods. We iteratively train the model on the training period of each fold. We then evaluate the RMSE on the validation period for each combination of hyperparameters, and we find the hyperparameters which minimize the RMSE on the validation period. Finally, we compute the RMSE on the test period using the optimal hyperparameters and we report the unweighted average RMSE across the test periods of the 10 folds.

A

	Expert	Lower bound	Upper bound	Traditional	Lower bound	Upper bound	Traditional + Expert	Lower bound	Upper bound	News	Lower bound	Upper bound	Expert +News	Lower bound	Upper bound	Traditional + News	Lower bound	Upper bound	Traditional + Expert + News	Lower bound	Upper bound
Full Model	0.1892	0.180	0.198	0.1486	0.126	0.168	0.1443	0.114	0.169	0.0990	0.079	0.115	0.0951	0.076	0.111	0.0819	0.051	0.104	**0.0817**	0.051	0.104
Afghanistan	0.1697	0.167	0.173	0.1329	0.09	0.165	0.1309	0.125	0.137	0.0940	0.071	0.113	0.0934	0.069	0.112	**0.0834**	0.074	0.092	0.0888	0.080	0.097
Burkina Faso	0.1586	0.081	0.209	0.1688	0.157	0.18	0.1323	0.109	0.152	0.1320	0.092	0.163	0.1309	0.09	0.162	0.1123	0.105	0.119	**0.1077**	0.101	0.114
Chad	0.1542	0.15	0.158	0.1276	0.115	0.139	0.1275	0.123	0.132	0.0980	0.085	0.109	0.0941	0.081	0.106	0.088	0.083	0.093	**0.0793**	0.074	0.085
Dem. Rep. of the Congo	0.1845	0.124	0.230	0.1824	0.171	0.193	0.1395	0.076	0.182	0.1350	0.131	0.139	0.0846	0.079	0.09	0.0847	0.041	0.113	**0.0785**	0.026	0.108
Ethiopia	0.1779	0.172	0.183	0.1447	0.133	0.155	0.1438	0.136	0.151	0.0940	0.086	0.102	0.0933	0.085	0.101	**0.0870**	0.054	0.111	0.0919	0.058	0.116
Guatemala	0.1845	0.176	0.193	0.1324	0.092	0.163	0.1323	0.129	0.135	0.0890	0.082	0.096	0.0833	0.076	0.09	**0.0720**	0.066	0.078	0.0750	0.069	0.081
Haiti	0.2900	0.283	0.297	0.1369	0.13	0.144	0.1274	0.122	0.133	0.0880	0.082	0.094	0.0835	0.077	0.089	0.0893	0.08	0.098	**0.0816**	0.071	0.091
Kenya	0.2100	0.202	0.217	0.1512	0.143	0.159	0.1505	0.147	0.154	0.1340	0.127	0.140	0.1254	0.118	0.132	**0.0930**	0.085	0.101	0.1174	0.110	0.124
Malawi	0.1755	0.168	0.183	0.1286	0.114	0.142	0.1276	0.121	0.134	0.0990	0.091	0.106	0.0973	0.089	0.105	**0.0831**	0.074	0.091	0.0918	0.084	0.099
Mali	0.2483	0.244	0.252	0.1499	0.104	0.185	0.1480	0.145	0.151	0.0850	0.077	0.092	0.0849	0.077	0.092	**0.0765**	0.028	0.105	0.0809	0.035	0.109
Mauritania	0.1879	0.160	0.212	0.189	0.172	0.205	0.1393	0.058	0.188	0.1383	0.027	0.194	0.0865	0.076	0.096	0.0897	0.064	0.109	**0.0760**	0.043	0.099
Mozambique	0.1852	0.124	0.231	0.1738	0.168	0.179	0.1576	0.145	0.170	0.1440	0.119	0.166	0.0854	0.025	0.118	0.0875	0.044	0.116	**0.0737**	0.070	0.077
Niger	0.1790	0.173	0.184	0.1397	0.081	0.18	0.1368	0.122	0.150	0.0990	0.098	0.100	0.0948	0.094	0.096	**0.0785**	0.068	0.088	0.0992	0.092	0.106
Nigeria	0.1770	0.168	0.186	0.1449	0.144	0.146	0.1419	0.134	0.149	0.0850	0.076	0.093	0.0840	0.074	0.093	0.0968	0.068	0.119	**0.0711**	0.013	0.100
Somalia	0.2920	0.287	0.297	0.1365	0.106	0.162	0.1352	0.123	0.146	0.0850	0.05	0.109	0.0847	0.048	0.110	**0.0813**	0.071	0.09	0.0833	0.073	0.092
South Sudan	0.1876	0.180	0.195	0.1466	0.114	0.173	0.1461	0.140	0.152	0.1350	0.122	0.146	0.1327	0.120	0.144	**0.0832**	0.055	0.104	0.1127	0.095	0.128
Sudan	0.1768	0.176	0.178	0.1459	0.133	0.158	0.1409	0.133	0.148	0.0870	0.053	0.111	**0.0827**	0.050	0.106	0.0836	0.074	0.092	0.0888	0.08	0.097
Uganda	0.1628	0.157	0.168	0.1587	0.121	0.189	0.1326	0.110	0.152	0.1302	0.097	0.157	0.1110	0.067	0.142	0.1180	0.112	0.124	**0.0747**	0.065	0.084
Yemen	0.1839	0.179	0.189	0.1332	0.072	0.174	0.1330	0.127	0.139	0.0920	0.083	0.100	0.0918	0.082	0.101	0.0876	0.08	0.095	**0.0837**	0.076	0.091
Zambia	0.1873	0.149	0.219	0.2111	0.201	0.221	0.1539	0.137	0.169	0.1529	0.136	0.168	0.0925	0.061	0.116	0.0885	0.079	0.097	**0.0766**	0.066	0.086
Zimbabwe	0.1456	0.134	0.156	0.1388	0.117	0.157	0.1288	0.123	0.134	0.0890	0.061	0.111	0.0843	0.053	0.107	**0.0778**	0.069	0.086	0.0811	0.072	0.089

B

	Expert	Lower bound	Upper bound	News	Lower bound	Upper bound	Expert + News	Lower bound	Upper bound
Full Model	0.1953	0.175	0.214	0.1698	0.157	0.182	**0.1684**	0.138	0.194
Angola	0.1994	0.187	0.211	0.1770	0.145	0.204	**0.1697**	0.147	0.190
Burundi	0.1861	0.164	0.206	0.1657	0.152	0.178	**0.1650**	0.143	0.184
Cameroon	0.1819	0.143	0.214	0.1598	0.127	0.187	**0.1469**	0.060	0.199
Central African Republic	0.1794	0.172	0.186	0.1619	0.132	0.187	**0.1598**	0.076	0.213
Congo	0.1968	0.168	0.222	0.1710	0.096	0.222	**0.1537**	0.068	0.207
Djibouti	0.1835	0.120	0.230	0.1666	0.098	0.214	**0.1635**	0.136	0.187
El Salvador	0.2137	0.177	0.245	0.1800	0.156	0.202	**0.1665**	0.133	0.194
Guinea	0.2577	0.243	0.271	0.2428	0.242	0.244	**0.2249**	0.215	0.234
Honduras	0.1818	0.169	0.194	0.1693	0.168	0.171	**0.1537**	0.138	0.168
Liberia	0.1937	0.160	0.222	0.1753	0.166	0.185	**0.1572**	0.149	0.165
Madagascar	0.1928	0.153	0.226	0.1769	0.176	0.178	**0.1624**	0.103	0.205
Rwanda	0.2390	0.213	0.263	0.1914	0.169	0.212	**0.1897**	0.168	0.209
Senegal	0.2168	0.210	0.224	0.1915	0.169	0.212	**0.1776**	0.169	0.186
Sierra Leone	0.1694	0.132	0.200	0.1595	0.084	0.209	**0.1566**	0.111	0.192
Tajikistan	0.1820	0.160	0.201	0.1634	0.162	0.165	**0.1569**	0.151	0.163
Tanzania	0.1874	0.154	0.216	0.1834	0.175	0.192	**0.1781**	0.153	0.200

C

Long term forecast	Expert	Lower bound	Upper bound	Traditional	Lower bound	Upper bound	Traditional + Expert	Lower bound	Upper bound	News	Lower bound	Upper bound	Expert +News	Lower bound	Upper bound	Traditional + News	Lower bound	Upper bound	Traditional + Expert + News	Lower bound	Upper bound
3 months	0.189	0.180	0.198	0.1486	0.126	0.168	0.1443	0.114	0.169	0.099	0.079	0.115	0.0951	0.076	0.111	0.0819	0.051	0.104	**0.0817**	0.051	0.104
6 months	0.193	0.165	0.218	0.1549	0.146	0.164	0.1517	0.134	0.168	0.117	0.114	0.120	0.1145	0.105	0.124	0.0968	0.096	0.098	**0.0962**	0.073	0.115
9 months				0.1713	0.155	0.186	0.1743	0.163	0.185	0.130	0.113	0.145	0.1340	0.098	0.162	0.1148	0.099	0.129	**0.1128**	0.097	0.127
12 months				0.1817	0.157	0.203	0.1818	0.180	0.184	0.142	0.133	0.151	0.1419	0.125	0.157	0.1390	0.134	0.144	**0.1375**	0.110	0.161

Fig. S8. Predicting food insecurity. (A) RMSE and 95% confidence intervals reported in Fig. 3A for the district-level predictions of the IPC classification of food insecurity three months ahead obtained from expert forecasts and from 6 random forest regression models estimated using observations from 21 countries over the period July 2009 to July 2020. We show the cross-validation RMSE and its 95% confidence interval on the entire test set (full model) and for each country separately. The lowest RMSE is shown in bold. (B) District-level predictions of the IPC classification of food insecurity three months ahead obtained from expert forecasts and from 2 random forest regression models estimated using observations from 37 countries over the period July 2009 to July 2020. We show the cross-validation RMSE and its 95% confidence interval on the test set observations from the 16 countries in which the traditional risk factors are unavailable from previous research (full model) as well as for each country separately. (C) Predictions of food

insecurity at 3, 6, 9 and 12-month horizons from expert forecasts – unavailable at 9- and 12-month horizons – and using random forest regressions estimated on the 21 countries for which expert forecasts, traditional and news factors are available over the period July 2009 to July 2020. We ensure that no observation from the training period is used to evaluate a model's performance. These results demonstrate that news indicators also improve the prediction of food insecurity up to twelve months ahead.

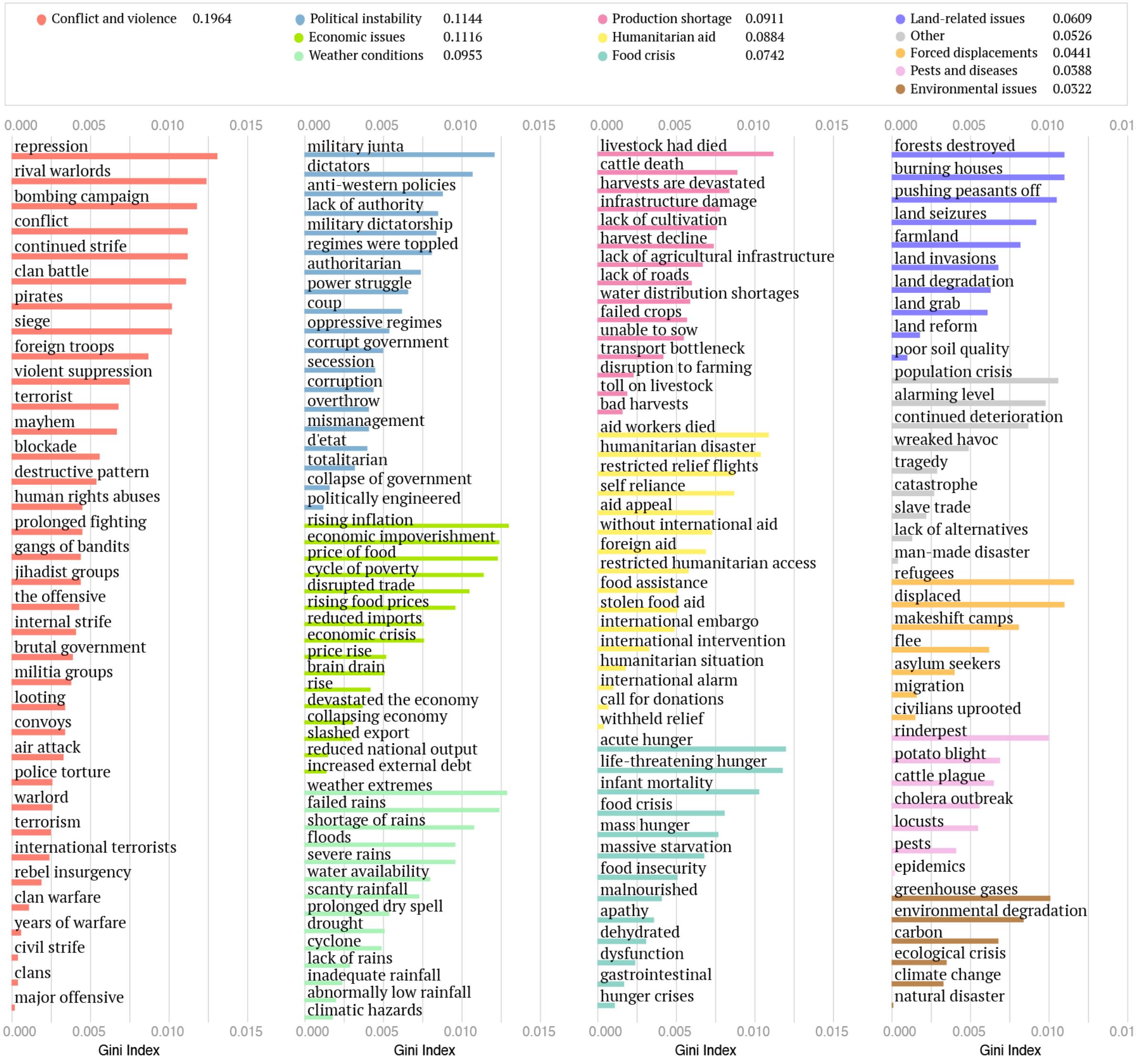

Fig. S9. Gini importance factors. We report the Gini index of the 167 news factors included in the traditional+news model. We also report the sum of the Gini index of all the features in a cluster, which indicates that the “Conflict and Violence” cluster provides the highest contribution to the predictions.

A

	Expert	Lower bound	Upper bound	Traditional	Lower bound	Upper bound	Traditional + Expert	Lower bound	Upper bound	News	Lower bound	Upper bound	Expert +News	Lower bound	Upper bound	Traditional + News	Lower bound	Upper bound	Traditional + Expert + News	Lower bound	Upper bound
RF	0.1892	0.1802	0.1978	0.1486	0.126	0.168	0.1443	0.1139	0.169	0.099	0.079	0.115	0.0951	0.0756	0.111	0.0819	0.051	0.104	0.0817	0.0506	0.104
RF + No z-scoring	0.1892	0.1802	0.1978	0.1483	0.137	0.159	0.1422	0.1307	0.153	0.095	0.065	0.117	0.0946	0.0812	0.106	0.0821	0.049	0.105	0.0819	0.0645	0.096
RF + Spatial autocorrelation	0.1892	0.1802	0.1978	0.1472	0.115	0.174	0.1442	0.1155	0.168	0.094	0.085	0.102	0.0935	0.0859	0.101	0.0815	0.080	0.083	0.0813	0.0710	0.090
RF + Food aid as input	0.1892	0.1802	0.1978	0.1481	0.136	0.160	0.1414	0.1321	0.150	0.094	0.073	0.111	0.0939	0.0671	0.115	0.0819	0.060	0.099	0.0816	0.0656	0.095
RF + No province and country aggregate values	0.1892	0.1802	0.1978	0.1532	0.145	0.161	0.1529	0.1430	0.162	0.106	0.084	0.124	0.1052	0.0857	0.122	0.0921	0.082	0.101	0.092	0.0794	0.103
LASSO (1949 news factors)	0.1892	0.1802	0.1978	0.1531	0.143	0.163	0.1526	0.1413	0.163	0.113	0.096	0.128	0.1128	0.1009	0.124	0.1002	0.077	0.119	0.1001	0.0781	0.118
LASSO (167 news factors)	0.1892	0.1802	0.1978	0.1531	0.142	0.164	0.1526	0.1399	0.164	0.118	0.098	0.135	0.1172	0.0981	0.134	0.1012	0.08	0.119	0.1009	0.0896	0.111
OLS (167 news factors)	0.1892	0.1802	0.1978	0.1521	0.125	0.175	0.1517	0.1375	0.165	0.100	0.089	0.110	0.0997	0.0882	0.110	0.0912	0.088	0.094	0.0911	0.0691	0.109

B

	RMSE	Lower bound	Upper bound	P-value
Traditional+News model	0.0819	0.0509	0.1040	
Only 13 seed keyphrases	0.1404	0.1217	0.1569	0.0001
No frame-semantic parsing of news	0.1271	0.1042	0.1464	0.0020
No frame-semantic parsing of books or journal articles	0.1077	0.0794	0.1300	0.0133
No keyword expansion	0.1192	0.0985	0.1368	0.0087
No dimensionality reduction	0.1246	0.1002	0.1450	0.0074
XG boost instead of Granger causality	0.1323	0.1308	0.1338	0.0008
Elastic net instead of Granger causality	0.0963	0.0796	0.1105	0.0120
Location within the same sentence as a text feature	0.0928	0.0609	0.1162	0.0351
Binary news indicators	0.1043	0.0760	0.1264	0.0261
No time-invariant factors	0.0846	0.0569	0.1052	0.0638
Including district-level article counts	0.0827	0.0558	0.1028	0.0785
Ablation of the largest news source	0.0953	0.0745	0.1123	0.0267
Ablation of the 10 largest news sources	0.1137	0.0928	0.1313	0.0051

C

	Expert	Traditional	Traditional + Expert	News	Expert +News	Traditional + News	Traditional + Expert + News
R^2	0.8215	0.8821	0.8901	0.8917	0.9526	0.9572	0.9873
Adjusted R^2	0.8201	0.8783	0.8896	0.8903	0.9488	0.9531	0.9812

D

	Model	Traditional	Std. Err.	Traditional + Expert	Std. Err.	News	Std. Err.	Expert +News	Std. Err.	Traditional + News	Std. Err.	Traditional + Expert + News	Std. Err.
AUC	>= 1 crisis period	0.7208	0.0031	0.7232	0.0032	0.8034	0.0241	0.8090	0.0064	0.8730	0.0069	0.8868	0.0055
	**>= 2 crisis periods**	**0.7317**	0.0081	**0.7348**	0.0031	**0.8158**	0.0065	**0.8231**	0.0062	**0.9025**	0.0062	**0.9112**	0.0047
	>= 3 crisis periods	0.7511	0.0032	0.7517	0.0013	0.8390	0.0048	0.8445	0.0027	0.9136	0.0062	0.9259	0.0056
	>= 4 crisis periods	0.7544	0.0079	0.7551	0.0051	0.8391	0.0030	0.8462	0.0090	0.9198	0.0063	0.9284	0.0051
	IPC<=2 to IPC>=4	0.7574	0.0076	0.7592	0.0051	0.8559	0.0058	0.8612	0.0085	0.9332	0.0075	0.9387	0.0050
Recall	Precision = 0.6	0.7151		0.7137		0.8353		0.8471		0.9281		0.9363	
	Precision = 0.65	0.7060		0.7091		0.8216		0.8344		0.9221		0.9310	
	Precision = 0.7	0.6824		0.6853		0.8079		0.8217		0.9161		0.9257	
	Precision = 0.75	0.6543		0.6572		0.7942		0.8089		0.9105		0.9204	
	**Precision = 0.8**	**0.6187**		**0.6374**		**0.7733**		**0.7966**		**0.9058**		**0.9151**	
	Precision = 0.85	0.5891		0.6014		0.7365		0.7544		0.8846		0.8858	
	Precision = 0.9	0.4691		0.5096		0.6119		0.6014		0.8333		0.8443	

Fig. S10. Robustness checks. (A) We compare the predictive performance of the random forest model presented in Fig. 3 with alternative specifications described in section S1.1 and S1.2. (B) We compare the predictive performance of the random forest model with traditional + news factors with the alternative specifications for the set of text features described in section S1.3-S1.5. (C) R-squared and adjusted R-squared of each model measured on the test set. (D) We report the area under the precision-recall curve (AUC) and its standard error (*34*) for each classification model of food crisis outbreaks (in column) and for different definitions of an outbreak (in row). We also report the recall of each model at different precision levels (in row). For both metrics, the row

showing our preferred specification is highlighted in bold. These results demonstrate that including news indicators consistently improves the traditional model's predictions.

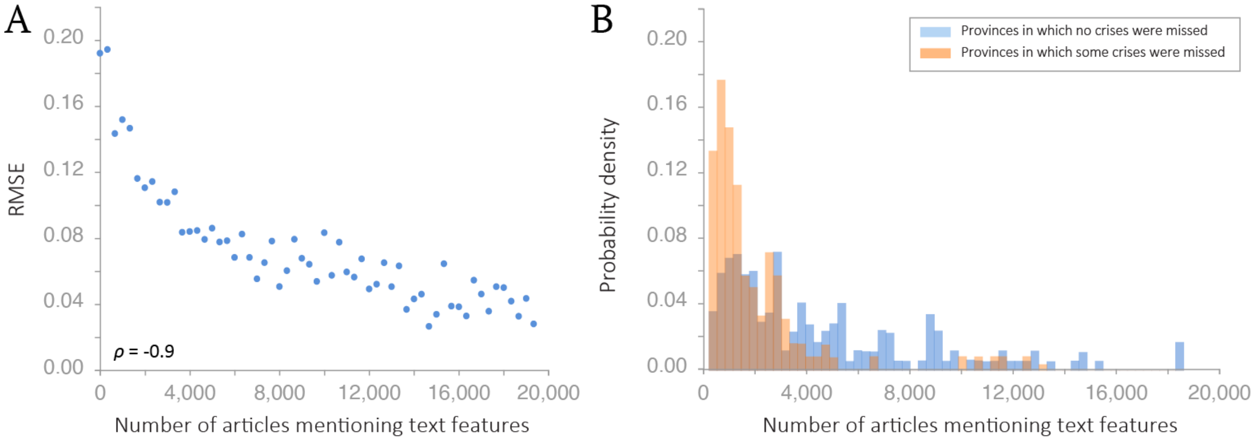

Fig. S11. News coverage and predictive performance. (A) The RMSE of a district is strongly negatively correlated (ρ=-0.9) with the number of articles mentioning text features and focusing on that district. (B) Distribution of the number of news articles mentioning text features across administrative units of level 1 ("provinces"), separating between provinces in which the traditional+news model predicts all the crisis outbreaks (blue) from those in which it fails to predict at least one crisis (orange), which reveals that provinces in which the traditional+news model fails to predict some crisis outbreaks have lower news coverage that those in which the model predicts all of them.
